# Behaviour in the workplace and monitoring data on occupational oral exposure to hazardous substances as prerequisites for the development of a model on occupational oral exposure: a systematic review and meta-analysis

**DOI:** 10.1093/annweh/wxaf015

**Published:** 2025-05-02

**Authors:** Marlene Dietz, Anke Kahl, Urs Schlüter

**Affiliations:** Unit 4.I.4 Exposure Assessment, Exposure Science, Division 4 Hazardous Substances and Biological Agents, Federal Institute for Occupational Safety and Health (BAuA), D-44149 Dortmund, Germany; Chair of Occupational Safety, School of Mechanical Engineering and Safety Engineering, University of Wuppertal, D-42119 Wuppertal, Germany; Chair of Occupational Safety, School of Mechanical Engineering and Safety Engineering, University of Wuppertal, D-42119 Wuppertal, Germany; Unit 4.I.4 Exposure Assessment, Exposure Science, Division 4 Hazardous Substances and Biological Agents, Federal Institute for Occupational Safety and Health (BAuA), D-44149 Dortmund, Germany

**Keywords:** oral exposure, ingestion, behaviour, occupational hygiene, measurement data, model, PRISMA

## Abstract

**Introduction:**

The assessment of the overall exposure of workers to hazardous substances is fundamental for their comprehensive protection. This includes oral exposure to hazardous substances, which can be relevant for total exposure depending on the specific workplace and substance. However, monitoring and available modelling approaches for a sound assessment of this exposure pathway are limited. The development of an occupational oral exposure assessment model requires knowledge of the contributing mechanisms, including worker behaviour and occupational hygiene practice. In addition, monitoring data on occupational oral exposure are a prerequisite for the evaluation of an exposure model.

**Methods:**

A systematic literature review was conducted using the PRISMA method. Studies describing behaviours and occupational hygiene practices with regard to oral occupational exposure, and studies including measured data sets for the model evaluation were identified. The included data were then extracted and evaluated in a meta-analysis.

**Results:**

142 studies addressing oral occupational exposure were identified in the area of behaviour and occupational hygiene. Frequencies of hygiene practices were aggregated. The influence of worker training was investigated and the controllability of individual behaviour was described qualitatively. For the model evaluation, 9 publications were identified that included monitoring data for oral occupational exposure. These publications use 5 different sampling approaches and describe 4 different substance groups.

**Discussion:**

The systematic literature search on behaviour and occupational hygiene provides a partly quantitative basis for the model-based description of occupational oral exposure to hazardous substances. Oral exposure to hazardous substances cannot be prevented by good occupational hygiene practices alone, as a relevant part of the causal personal behaviour (eg touching the own face) is subconscious and can therefore not be avoided completely. The identified database serves as a basis for the later model evaluation. The usability of the data sets depends on whether the future model input parameters are documented in the studies.

**What’s Important About This Paper?**

Oral exposure at workplaces is only rarely addressed during occupational risk assessments, though it is relevant in many workplaces. This study reports a systematic literature review of relevant behaviours and occupational hygiene practices as contributing mechanisms to oral exposure, and summarizes available measurement data. The data in this paper provide a basis for model development and evaluation related to occupational oral exposure.

## Introduction

For the protection of workers’ health, the assessment of their exposure to hazardous substances is crucial (Fantke et al. 2022). When measurements are not feasible due to lack of available measurement methods or ethical aspects of measurements in human subjects, modelling is an essential assessment method (Schlüter et al. 2022). According to European chemical legislation (eg The European Parliament and the Council of the European Union 2006, 2012) and Occupational Safety and Health (OSH) requirements (Committee on Hazardous Substances (AGS) 2017), all routes of exposure must be included in the worker exposure assessment to ensure comprehensive protection. Nevertheless, some exposure pathways have been studied little so far.

In a systematic literature review and meta-analysis, Dietz et al. (2023) investigated the relevance of occupational oral exposure and concluded that it can contribute significantly to total worker exposure, especially in indoor, industrial, and recycling workplaces (Dietz et al. 2023). Furthermore, this work summarized that, in general, different monitoring and modelling approaches are documented. This work is based on and complemented by several studies, which indicate that oral exposure is a relevant route in the workplace (Karita et al. 1997; Cherrie et al. 2006; Christopher 2008; Gorman Ng et al. 2016; Sinclair et al. 2016; Beattie et al. 2017; Cherrie 2023).

Our goal is to develop a comprehensive understanding of unintentional oral exposure in various types of workplaces to enable the development of a practical model for quantitative estimation of this pathway. For example, workers in a lead refinery were orally exposed to lead deposited on their face and under their fingernails (Karita et al. 1997) and workers in e-waste dismantling can ingest organophosphate esters due to hand-mouth contacts (Zhang et al. 2021). In particular, information is needed on the mechanisms contributing to occupational oral exposure, such as occupational hygiene practices and worker behaviour. This could be, for example, the frequency of hand washing during a shift or contact between the hand and the region close to the mouth, the so-called perioral region. The available qualitative information on behaviours and occupational hygiene practices influencing oral exposure is limited in the scientific literature. Analogously, quantitative data on hygiene practices and relevant behaviour is rare. Qualitative and quantitative information on relevant behaviours is especially limited for adults and workers.

Consequently, it is important to synthesize and evaluate the available limited information. Therefore, we conducted a systematic literature search and meta-analysis focussing on behaviour and hygiene at workplaces that might influence oral exposure to hazardous substances.

In addition to understanding the contributing mechanisms of occupational oral exposure, the development of an appropriate exposure model requires its evaluation by comparison to data. An additional systematic literature search and meta-analysis was performed to identify appropriate measurement data sets that can be used to conclude on occupational oral exposure.

## Methods

For the analysis of relevant behaviours and hygiene practices, the following two main questions were raised:

1. How often do relevant behaviours, eg hand to perioral contact, occur in adults?
2. How often are relevant occupational hygiene practices and measures performed in the workplace, e.g. hand washing?

To further contextualize this research, the following two additional questions were posed and evaluated:

3. According to OSH (DGUV 2013; Federal Ministry of Labour and Social Affairs Germany 2023), trainings must be conducted on a regular basis. Is this successful in improving worker compliance with good occupational hygiene practice? Is the database sufficient to cover this as a model input parameter?
4. Each individual must personally change their behaviour to minimize the occurrence of occupational oral exposure. Is it possible to consciously avoid behaviours that contribute to oral exposure?

An additional search was conducted on between and within worker variability as behaviour-related topic. However, this is out of scope of this publication and therefore not presented.

As our long-term goal is to develop a model for occupational oral exposure, a fifth question was raised in a second literature review:

5. Which measurement data is documented in the literature for occupational oral exposure and might be used in a first model evaluation?

The two systematic literature reviews were conducted using the PRISMA method (Page et al. 2021). No study protocols were defined or registered for either review.

### Information sources and search strategies

As information sources, both reviews rely on PubMed, Web of Science, COCHRANE, Deutsche Nationalbibliothek, and bergischbib as databases. The behavioural review additionally includes PubPsych as a sixth database to cover its focus on behaviour and consciousness.

In addition, the websites of 9 research institutes were searched for both reviews to include other relevant publications not already included in the databases. A summary of all databases is provided in Supplementary Table 1.

Detailed search terms have been defined to search these databases and websites. These are usually in English and based on several synonyms covering the relevant topics. However, most of the websites do not necessarily allow complex search strings, so that simplified strings and concretizing word groups were developed. In addition, most of the included publications in the databases “Deutsche Nationalbibliothek” and “bergischbib” are in German, so analogous German synonyms were used.

One search strategy was developed for each of the research questions of the two reviews, resulting in 5 strategies. The topics, covered by synonyms are shown in Table 1, while the detailed search strategies are summarized in Supplementary Table 2.

**Table 1.** Topics covered by multiple synonyms in individual search strategies and results of the evaluation of search strings.

Question	Topics covered by multiple synonyms	Identified/available pre-known publications	
		**Web of Science**	**PubMed**
1. Relevant behaviour	workers/adult study participants; contact frequencies/behaviour; perioral region/face; hands	7/7	7/7
2. Hygiene practice	Employees; hand washing/occupational hygiene; the survey method to ensure the availability of concrete data. Exclusion: hand washing as a sampling method for dermal exposure	5/5	4/5
3. Trainings	Employees; occupational hygiene; trainings; their effect	1/1	1/1
4. Consciousness	Touch; face; awareness	1/1	1/1
5. Measurement data	Oral exposure/measurement methods; occupation; measurement Exclusion: Medicine, animal testing; smoking cessation; lip seal	4/4	4/4

The specified topics and synonyms were optimized to maximize the number of identified relevant publications while minimizing the number of irrelevant publications. For this iterative optimization, publications with relevant content were identified manually in advance. These pre-known publications were then used to test whether the developed search strategies identify relevant and suitable publications. The corresponding test results are documented in Table 1. Detailed information on the used publications, their availability in the databases and the evaluation results of the individual search strategies are given in Supplementary Tables 3 and 4. The applied search strategies and dates of last search are summarized in Supplementary Tables 2 and 5.

### Study selection

Once the search strings have been used to identify publications, the suitability of these publications must be assessed. Therefore, a two-step screening of publications was performed. First, the suitability was assessed based on title and abstract. Second, the remaining full texts were screened. Both steps require concrete criteria that can be reproducibly applied to multiple publications. Therefore, the criteria were divided into Population (P) and Outcome (O) criteria for each screening step, as summarized in Supplementary Table 6.

The number of relevant studies on the behaviour of workers is very limited. Therefore, if possible, adults were selected as the Population for both screening levels, rather than including only worker-specific information. It was assumed that the behaviour of children differs significantly from that of adults, so publications that only provide information on behaviour of children were excluded.

The Outcome criteria are defined individually for the different search strategies and become more detailed for the second screening stage based on full texts as more information becomes available to assess eligibility. In addition, the criteria attempt to ensure the exclusion of studies that do not fit the focus of the reviews. For example, studies on training effectiveness should focus on aspects relevant to the emergence of oral exposure, ie on behaviour. In contrast, training on specific work techniques, eg welding, would not be relevant.

Additionally, the searches were limited to publication years between 2000 and 2023. Publications had to be in English, with the exception of the databases Deutsche Nationalbibliothek and bergischbib, where German language was accepted.

To assess the suitability and reproducibility of these criteria, consistency checks were performed for both screening stages. The criteria were applied in parallel by two researchers to 50 and 10 publications for the title abstract and full text screening, respectively. The agreement of the ratings was formally analysed by calculating Cohen’s kappa, which evaluates the consistency under consideration of the consistency that would be expected by chance (Warrens 2015). The results and criteria were discussed, the wording of the criteria was sharpened, and inconsistencies were resolved by consensus. The screening was then performed by MD.

For the automatic removal of duplicates, CADIMA 2.2.4.2 (Kohl et al. 2018) was used. Further duplicates were deleted manually, eg different abbreviations of journal names could not be determined automatically. Furthermore, consistency checks and both screening steps were performed in CADIMA.

### Data extraction and data analysis

The relevant information was then extracted from the publications. Concept matrices were used for this purpose because these tables contain detailed categories that are designed to represent the complex information in the publications (Webster and Watson 2002). For example, “A mean value for the frequency of hand-mouth contact is available” would be a detailed concept to categorize whether this information is available in individual publications. This concept would not only be checked, but the concrete value would be entered here to prepare the further quantitative evaluation. This approach is an extension of the classical concept matrix. In this way, a clear and structured overview of complex qualitative and quantitative information was achieved. This allows the further processing of the information and data from multiple publications.

For the behavioural and occupational hygiene review, information was structured according to the 4 different search strategies. Within each search strategy, information was categorized according to, eg different contact types such as hand-mouth or object-mouth, different occupational hygiene measures, training formats or conclusions on consciousness. In addition, different types of statistical descriptors such as medians or means were distinguished, when available. Similarly, a categorization was developed for the review of measurement data, distinguishing between sampling types and substance groups.

Qualitative conclusions were assessed according to their frequency in different publications. Quantitative data were evaluated individually, with the aim of preparing these data for later use in a development or evaluation of a model for occupational oral exposure assessment.

Because multiple study designs were analysed, which are often unregistered, a quantitative assessment of bias was not possible. Instead, a qualitative discussion of bias was conducted.

## Results

### Study selection and included studies

For the behavioural review, Cohen’s kappa was calculated to be 0.17 and 0.50 for title abstract and full text screening, respectively. This corresponds to slight and moderate agreement according to Landis and Koch (Landis and Koch 1977). The reasons for disagreements were discussed between the two reviewers and misunderstandings regarding the application of criteria were identified. For example, employees should be trained themselves and not offering training to others. The accordingly improved criteria are those summarized in Supplementary Table 6. The discussion also revealed, that the criteria themselves were well suited, despite the low kappa values. The reviewers agreed that the clarified wording was sufficient to ensure a consistent evaluation of the publications.

For the measurement data review, Cohen’s kappa was 0.60 and 0.63 for title abstract screening and full text screening, respectively. According to Landis and Koch (Landis and Koch 1977), this corresponds to moderate or substantial agreement. Therefore, the reviewers discussed and agreed on slightly sharpening of the criteria, as summarized in Supplementary Table 6.

Based on the consistency checks, the finalized criteria were applied to both reviews and screening levels.

A total of 6,083 publications (including 241 on variability, not presented here) were identified for the behavioural review. Of these, 4,504 publications remained after duplicate removal. Title abstract screening and full text screening excluded 3,848 and 514 publications, respectively. Finally, 142 (including 52 on variability, not presented here) publications were included for further data extraction.

The measurement data review identified 1,851 publications, of which 1,599 remained after duplicate removal. The title abstract screening and the full text screening excluded 1,572 and 18 publications, respectively, resulting in 9 included studies.

**Fig. 1.**
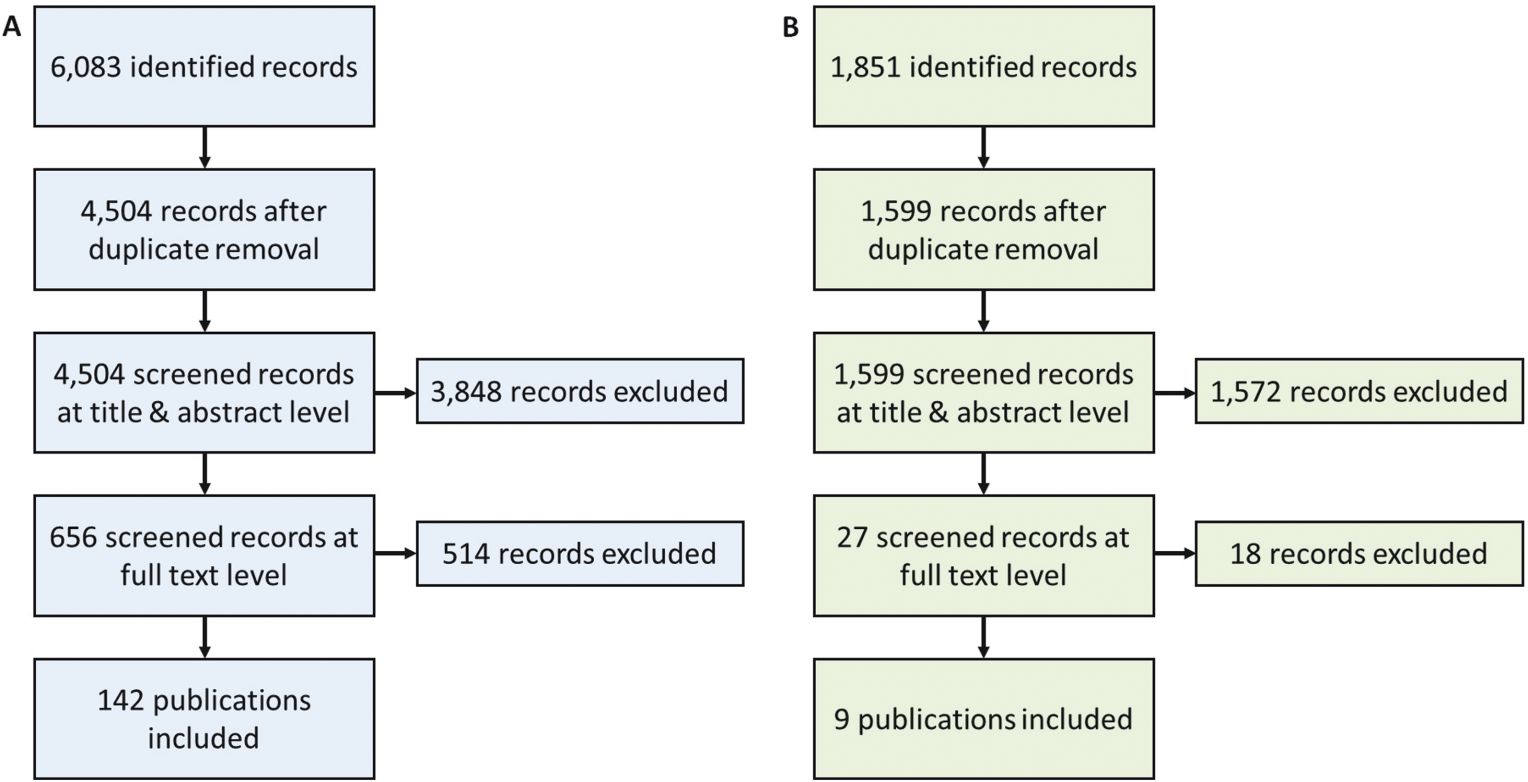
PRISMA flow diagram of the review on (A) behaviour and occupational hygiene practice and (B) measurement data for a later model evaluation.

Both screening processes are summarized in the flowcharts in Fig. 1. A summary of the included publications and their included information by search strategies is given in Supplementary Tables 7 and 8.

Based on the identified publications, categories were developed to ensure a sound evaluation of the information included. This evaluation focussed, eg on frequencies of different contacts, consciousness of contacts or investigated substances and sampling methods. A summary of the detailed categories is shown in Supplementary Fig. 1 for both reviews.

### Contact frequencies

The contact frequencies information was extracted from 18 publications, which describe indoor workplaces (number of publications *n* = 10, eg offices or lecture halls), nursing (*n* = 3), laboratories (*n* = 3), other industrial workplaces (*n* = 2), metal working (*n* = 2), power plants (*n* = 1), outdoor workplaces (*n* = 1), agriculture (*n* = 1), pest control (*n* = 1), and workshops (*n* = 1). The respective studies are itemized in Supplementary Table 7. The number of observed adults varies between 9 and 1,090 persons, with an average of 99. Therefore, a broad range of workplaces and personal habits is covered resulting in data which can be used for the evaluation of oral exposure.

The publications report the information on different contact frequencies most often as a mean or median value, as a maximum value or as a range. In addition, the types of contact described varied widely. The most common types of contact described were between hands, arms and objects on the one side and the face or mouth on the other side. Additionally, in some publications, the face and mouth regions were subdivided. For the evaluation in this meta-analysis, the category “face” therefore also includes data on the T-Zone which summarizes the forehead, nose and chin. The category “mouth” summarizes contacts described as either “mouth,” “oral / oral cavity,” or “perioral.” Based on this definition, contacts with the mouth, including the perioral area, are more likely to contribute to oral exposure than contacts with the face, excluding the perioral area.

**Fig. 2.**
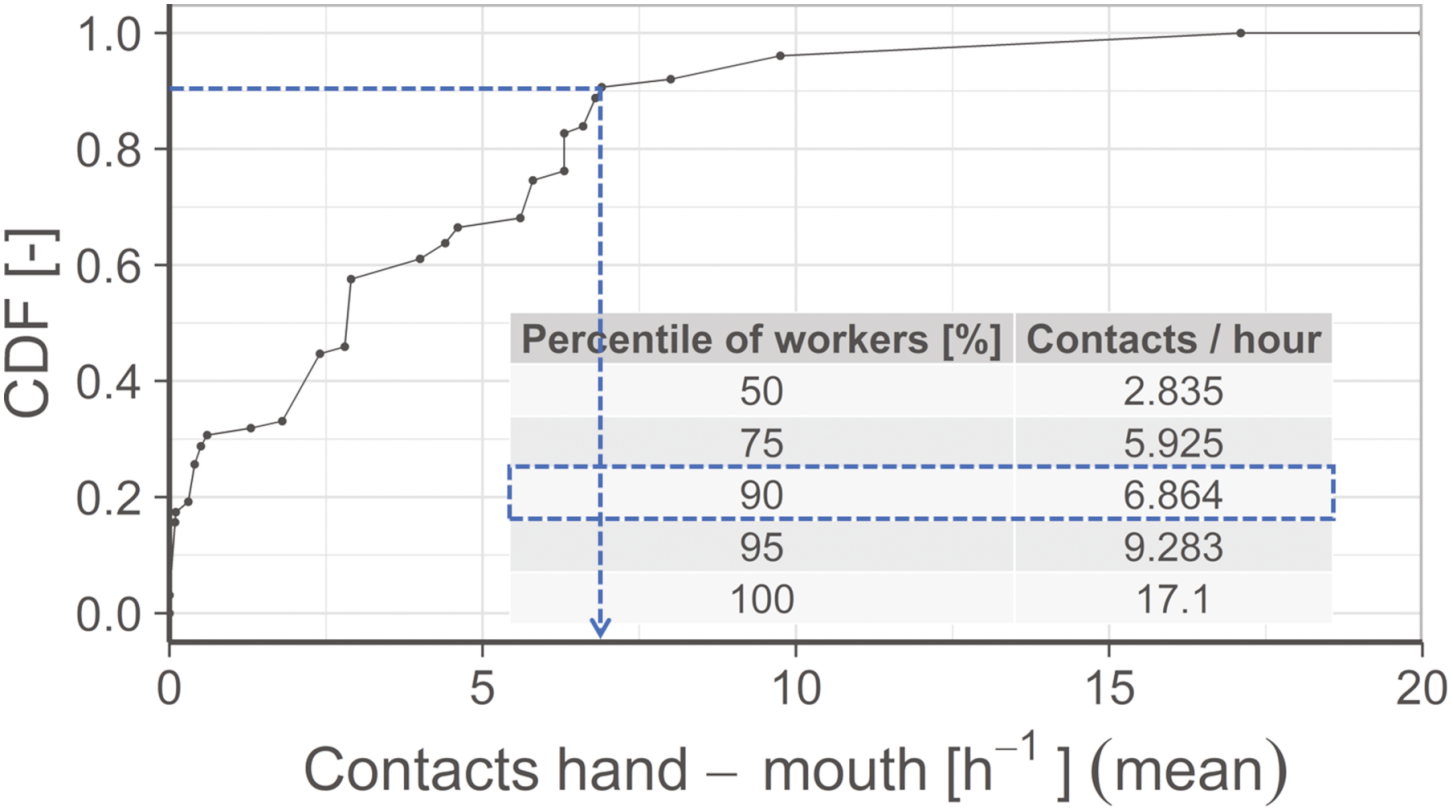
Cumulative distribution of hand-mouth contact based on published mean values and corresponding percentile values based on number of workers. The arrow illustrates the example for 90 % of the workers. The Poisson distribution for hand-mouth contacts per hour follows λ = E(X)=Var(X) = 3.836 h^-1^.

A description of the frequency of contributing behaviours is necessary to describe the emergence of occupational oral exposure in a model. The data indicate that these contact frequencies are individual-based and therefore vary considerably. Thus, it is essential to be cautious when selecting the frequencies of behaviours to be included in a model. We propose a cumulative distribution based on the number of workers performing specific contact frequencies. This allows a differentiated selection of a percentile of workers, which should be represented in a specific model calculation. Therefore, the extracted data from all publications were aggregated according to their data type and the observed contact category. Then, cumulative distributions were created based on the number of workers from all publications, for which the corresponding contact frequency was observed. An example for hand-mouth contacts is illustrated in Fig. 2. This distribution shows that 90% of the observed workers do not perform more than 6.9 hand-mouth contacts per hour, as illustrated in Fig. 2.

Additionally, qualitative information about influencing factors was extracted. Stress, personal habits such as nail biting, anxiety or nervousness are some of the personal factors (Cherrie et al. 2006) that have an influence on the observed contact frequencies. Personal protective equipment, such as gloves or respiratory protective equipment, can influence behaviour, as well as the duration of breaks (Gorman Ng et al. 2016; Shiraly et al. 2020). Awareness of the hazards posed by substances can lead to a reduction in the frequency of contact of hands to face (Nicas and Best 2008).

The extent to which the work is dominated by manual tasks, and thus, the extent to which the hands are not available for mouth or face contact, is a factor with a direct influence on contact frequency (Cherrie et al. 2006; Christopher 2008; Gorman Ng et al. 2016). Sitting as a posture is also increasing contact frequency due to the spatial proximity of hands and face (Christensen et al. 2021).

### Occupational hygiene practice

The included studies on occupational hygiene practices focus on nursing (*n* = 16), indoor workplaces (*n* = 8), food sector (*n* = 5), other industrial workplaces (*n* = 4), agriculture (*n* = 3), laboratories (*n* = 1), and workshops (*n* = 1). The respective studies are itemized in Supplementary Table 7. The number of participants in studies on quantitative information on hygiene practices ranges from 12 to 9,051 with an average of 358.

Information on occupational hygiene practices could be collected for handwashing, hand disinfection and glove wearing. The frequency of handwashing or hand disinfection was mainly documented as intervals, eg 5 workers wash their hands 0 to 4 times per day, 3 workers wash their hands 5 to 7 times per day, and 2 workers wash their hands at least 8 times per day. To summarize these intervals, weighted means $\bar{x}$ were calculated as described in Supplementary Section 2.

**Fig. 3.**
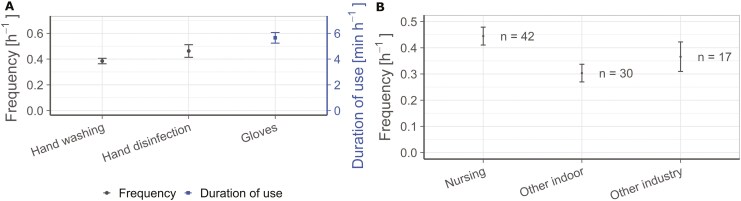
(A) Frequencies of hand washing and hand disinfection (left axis). Wearing duration of gloves (right axis). (B) Aggregated frequencies of handwashing according to different groups of workplaces. All values as weighted means with error bars based on Gaussian error propagation.

Figure 3A (left axis) presents the resulting weighted means for the frequency of handwashing and hand disinfection. The error bars result from a Gaussian error propagation (Supplementary Section 2) that takes into account the uncertainty of the frequency intervals rather than exact frequency values. Similarly, the duration of glove use in the studies were processed (Fig. 3A, right axis), as most frequently this information is given rather than a frequency of glove wearing. Even though glove changes cannot be interpreted based on this information, potential behaviour changes due to glove wearing could still be included in a future modelling approach based on this temporal information.

According to the data, hand disinfection occurred more frequently than hand washing. Gloves were worn for more than 5 min per hour. Taking these results into account, the underlying workplace groups were analysed as visualized for hand washing in Fig. 3B. The hand washing frequency in the food sector is not visualized, as only two data points were available.

The frequencies of handwashing, hand disinfection and glove use have been studied more among nurses than in other occupations. Therefore, the database may not represent other workplace groups in terms of frequency of hand washing and disinfection and duration of glove use.

Also, data on efficiencies of hygiene measures were collected. There are 20 and 44 values for the efficiency of hand washing and hand disinfection, respectively. However, each value corresponds to very specific conditions of use, such as soap, duration of application, etc., and therefore, no overall or summary values were calculated.

Some publications recommended qualitatively the reading of chemical labels, covering containers to avoid splashes, and using special soaps to reduce occupational oral exposure (Blanco et al. 2005; Hasylin et al. 2022).

### Influence of trainings

Included studies describing the influence of trainings in the workplace focus on nursing (*n* = 14), agriculture (*n* = 5), food sector (*n* = 4), outdoor workplaces (*n* = 2), indoor workplaces (*n* = 2), construction sites (*n* = 1), metal working (*n* = 1), and other industrial workplaces (*n* = 1). The respective studies are itemized in Supplementary Table 7. The number of study participants varies between 20 and 500, with an average of 96.

In a future model, training on behaviour might be an input parameter affecting the assumed relevant behaviour. The effectiveness of training was calculated by analysing the impact of training in the workplace on behaviour or general occupational hygiene practice. The approach of such studies is usually as follows: in a first step, the workers knowledge is assessed in an exam. Alternatively, the compliance of workers behaviours, eg with rules for hand disinfection, is assessed by an observation. In a second step, the training will be conducted. Third, an analogue observation or exam is performed to assess the behaviour or knowledge after the training. By comparing the number of compliant behaviours, or the correct answers in exams before and after the training, the efficiency is calculated. Data were collected on a variety of training formats, including online or face-to-face, poster, hands-on, video, and other methods (eg hand-outs, composition of songs or mnemonics, e-learning games). Depending on the training format, the medians of these efficiencies ranged from 16% to 31%.

**Fig. 4.**
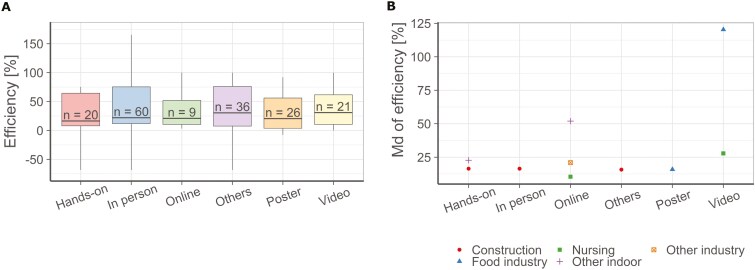
(A) Boxplot of training efficiencies for different training formats. Outliers are not plotted for better readability. Horizontal lines represent the 25^th^ percentile, the median and the 75^th^ percentile. Whiskers represent the minimum and maximum values in the data greater than the 25^th^ percentile—1.5 * IQR or less than the 75^th^ percentile + 1.5*IQR. (B) Median efficiency values for training formats differentiated for workplace groups.

A boxplot (Fig. 4A) of the obtained efficiency values was generated to illustrate the range of efficiencies per training format. The studies show a large variability in efficiencies because the knowledge base varies widely. Therefore, the same training design can lead to large or small improvements compared to the baseline. Furthermore, there were no major differences between different training format efficiencies.

Similarly, training efficiencies were considered separately for different groups of workplaces to evaluate whether this allows more specific conclusions on the influence of trainings. In that case, these conclusions could flow into future modelling approaches for occupational oral exposure. Therefore, the groups of workplaces were distinguished as shown in Fig. 4B. In particular, online training for indoor workplaces and video-based training in the food sector with more than 50% and more than 100% improvement, respectively were more efficient than other training formats. However, based on the available data, there is still no clear trend in the evaluation of efficiency for neither workplace groups nor training formats which could be used in the modelling of occupational oral exposure.

In a future model, training might be taken into account as a modifying factor with only a small influence to conservatively cover the wide range of efficiencies. Such a factor will not distinguish training formats, as the analysis does not allow any distinction.

### Controllability of behaviour

Especially publications from brain research describe spontaneous contacts between hand and face as performed with little or no awareness (Grunwald et al. 2014; Mueller et al. 2019). Different studies investigated relevant contact behaviour and documented influencing factors as well as frequencies of (sub-)conscious behaviour.

Twelve publications stated that behaviours such as hand-face contact may be subconscious. Three studies supported this statement by describing these behaviours as (partially) uncontrolled. On the other side, two studies describe these behaviours as controlled. However, one study later invalidated this point of view. Ten publications considered behaviours as hand-face contact as necessary for self-regulation in emotionally or cognitively demanding situations.

Consequently, behaviours that may contribute to oral exposure are not necessarily conscious. In addition, if the behaviours are necessary for self-regulation, it cannot be assumed that these are completely avoidable in the workplace.

Quantitatively, the mean frequency of subconscious hand-face contacts from the included studies was 17.31 per hour. In particular, this mean value is based on studies that describe artificial situations designed to place participants intentionally under cognitive or emotional load, eg by giving them challenging cognitive tasks while playing loud and distracting sounds (Mueller et al. 2019; Spille et al. 2022). Compared to the cumulative distribution of all hand-face contacts without intentionally increased emotional or cognitive load, only about 5 % of workers touched their face more than 17.31 per hour (Supplementary Fig. 2). Therefore, contact frequencies from studies with intentionally increased emotional or cognitive load may provide reasonable worst-case values for demanding work situations.

### Identified database for model evaluations

Nine studies were identified in the review of measurement data for an evaluation of an occupational oral exposure model. The studies describe metal working (*n* = 7), agriculture (*n* = 2), nursing (*n* = 1), and workshops (*n* = 1) (Supplementary Table 8). The included measurements are most commonly based on perioral wipes as shown in Supplementary Table 9. Other sampling methods identified were saliva sampling/mouth rinses, lip wipes, fingernail scrapings, or other biomonitoring.

Metals were the most frequently evaluated group of substances. Water, pesticides and pharmaceuticals were also investigated in the publications, as summarized in Supplementary Table 9.

**Fig. 5.**
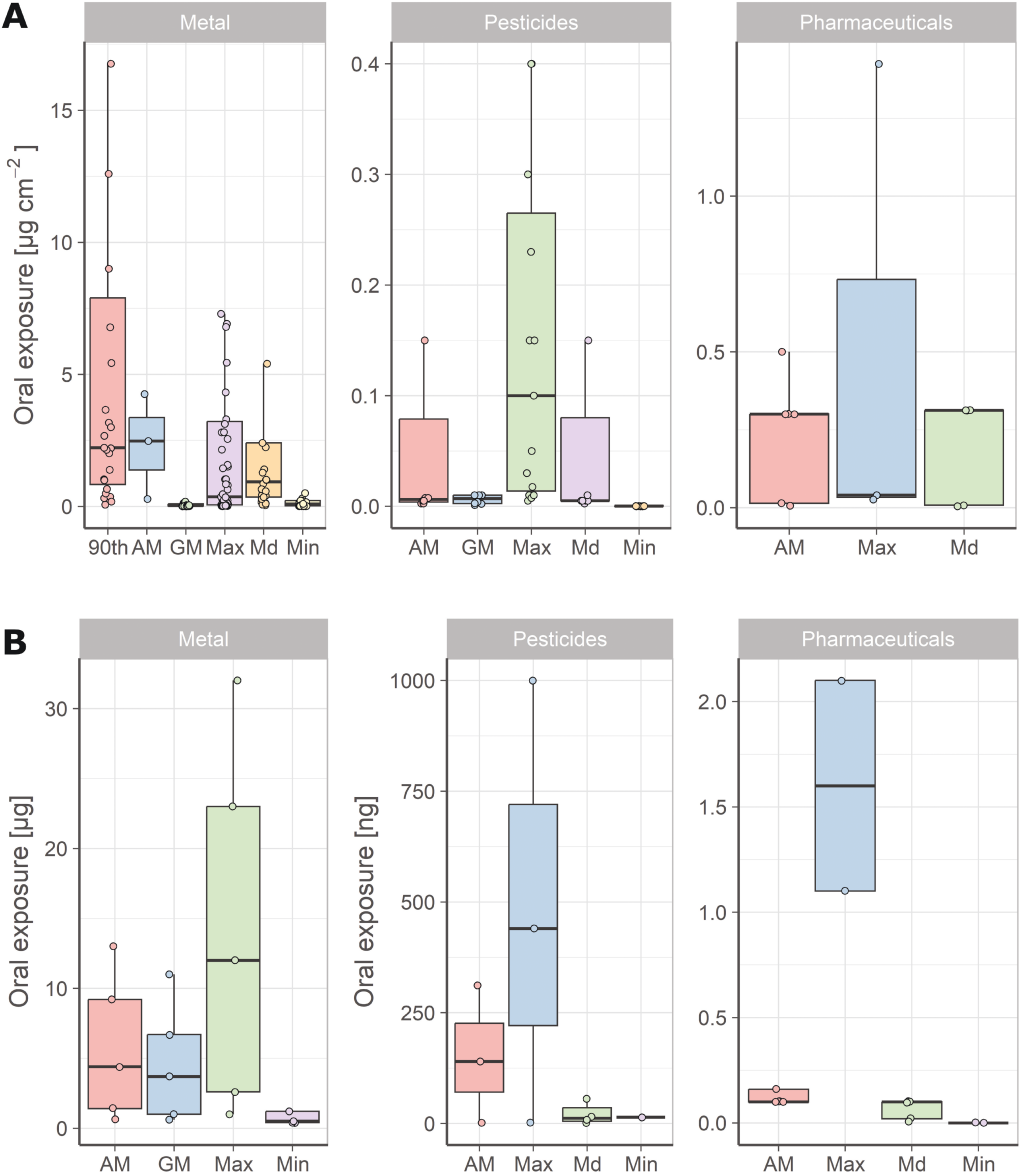
Boxplot of the available measurement data on oral exposure based on (A) Perioral (wipes) and (B) Saliva sample/mouth rinse. Outliers are not plotted for better readability. 90th: 90th percentile, AM: arithmetic mean, GM: geometric mean, Max: maximum, Md: median, Min: minimum.

Boxplots were prepared to get better insight in this database for the single sampling methods distinguishing substance and type of available statistical information (eg geometric mean, median, or maximum). Data points were overlayed to better represent small databases, although outliers were not included. Figure 5A and B include the database for perioral wipes and saliva samples/mouth rinses, respectively. Caution needs to be paid as different units were used in the literature depending on the sampling method. Thus, results from different methods cannot directly be compared. Within both the perioral wipe and the saliva/mouth rinses results, metal samples resulted in higher oral exposure estimates than pesticides or pharmaceuticals.

The boxplots for the other sampling methods can be found in Supplementary Section 4, although the corresponding databases are limited.

The applicability of these studies for the evaluation of an occupational oral exposure model depends on the specific application and input parameters of the respective model, and on the scenarios and parameters (as inputs for the model) documented in the studies.

### Analysis of bias

Bias can occur for a variety of reasons, including non-reporting of nonsignificant results and misinterpretation of a lack of research as a lack of importance.

The limited adult contact frequency database may introduce bias, by not focussing on potentially relevant types of contact, thus distorting the overall picture of relevant contact types and their frequencies.

Studies for general occupational hygiene practices have been conducted primarily in the nursing context. Other studies were available for workplaces in industrial, indoor, and food sector workplaces. As there are many other workplace groups, the pooled data must be interpreted with caution, as there is a bias towards the few different underlying workplace groups.

A wide range of studies was identified regarding the studies on training efficiency. These studies have different characteristics, such as prior knowledge, which cannot be adequately compared for the different studies based on the available information. Therefore, it cannot be determined whether these differences lead to a bias. In addition, observational studies of training efficiency may be influenced by the Hawthorne effect (York et al. 2012; Roberts et al. 2023), which describes the difference in behaviour of observed individuals compared to their natural behaviour, and so also introduces bias.

In the field of consciousness research, the recent focus has been on hand-face contact and its interplay with emotional and cognitive challenges. However, there may be other types of contact and influences on them, such as the availability of hands during manual work that have not been focussed on in this context. Additionally, the Hawthorne effect might affect observational results on consciousness as well. Thus, there may be aspects of consciousness that need to be further assessed to gain a comprehensive and less biased impression of this field and its importance for oral exposure.

The results of the second review on measurement data of oral exposure summarized that most measurement data are available for the substance group of metals. This could lead to a bias if it is assumed that other groups of substances do not need to be further evaluated, which contradicts the results of Dietz et al. 2023 (Dietz et al. 2023).

## Discussion

The identified data provide a quantitative description of adult contact frequencies based on percentiles, which can be used for model-based assessments of occupational oral exposure. However, there was limited coverage of different types of contacts, such as contacts with objects and arms, and the database for adults, especially for workers, is still very limited.

In terms of occupational hygiene practice, reference values could be aggregated, but these are only focussed on work in the healthcare sector. The weighted mean values for hand washing and hand disinfection in the nursing context were 3.56 and 4.56 events per 8 h shift, respectively. Compared to this, HEAdhoc-Recommendation 9 on hand disinfection in hospitals assumes 10 hand washing and 25 hand disinfection events per shift (BPC Ad hoc Working Group on Human Exposure of the European Chemicals Agency 2017). These numbers are estimated based on work flow, patient number per nurse and the resulting theoretical number of disinfections between different patient contacts. The HEAdhoc-Recommendation 9 leads to higher estimates than the observations in this study. Hygiene practices in other sectors may differ due to less strict regulations and a smaller awareness of hygiene requirements. In the future, assessments of occupational hygiene practice in other sectors will be needed. Based on these data, a comparison of different sectors, including the healthcare sector, would be of interest to analyse the transferability of the available information. Another topic for comparison and analysis of long-term influences is the change of hygiene practices due to the COVID-19 pandemic. In this review, 7 of 37 studies on hygiene practice were published during or after the pandemic giving a starting point for future analysis.

The medians of the training efficiencies for the identified training formats ranged from 16% to 31%. However, success factors of trainings are not yet deeply investigated, especially in other sectors than healthcare. In-depth knowledge of the effectiveness of training and the relevant influencing factors would be required, if the effects of training should be taken into account in a differentiated manner in modelling approaches.

The results on the controllability and consciousness of hand-face contact, which can contribute to occupational oral exposure, give an important indication that these behaviours cannot be neglected in the workplace. Nevertheless, the distinction of conscious/controlled and subconscious/uncontrolled behaviour can be challenging. This will lead to uncertainties in the results and underlying studies might have varying definitions of the regarded increased emotional or cognitive load. In the future, influences that can reduce emotionally and cognitively necessary contacts could be identified to target their reduction.

Nine identified studies provided measurement data that can potentially be used to evaluate an occupational oral exposure model. Dietz et al. (Dietz et al. 2023) described a focus of recent research on the substance group of metals, which is also represented in these studies, as 7 (out of 9) studies include measurements of metals. Therefore, it must be critically assessed which of these studies can be used for the evaluation of a model, as measurement specifications and model characteristics must be compatible.

### Limitations and strengths

Both systematic reviews were limited to studies published from 2000 to 2023 and to studies published in English. Nevertheless, it is assumed that most of the relevant studies were identified, and the behavioural review in particular is very comprehensive. Only one of the authors (MD) screened the publications at title abstract and full text level. However, clear criteria for Population and Outcome were defined in advance for both screening stages. The criteria were evaluated in consistency checks, to ensure a reproducible and self-consistent assessment during the screenings. Similarly, data extraction was performed by only one of the authors (MD). To ensure consistent and correct extractions, relevant text passages were extracted for documentation. The information was extracted according to clearly defined and detailed categories, as documented in the concept matrix.

A strength of both reviews is the definition of comprehensive and detailed search strategies. Especially for the behavioural review, a separation into different research questions was implemented and reflected in detailed search strategies. This allowed direct coverage of workplace behaviour and occupational hygiene practice. The results were supported by further in-depth searches on training, and controllability. Based on the systematic identification of information and the subsequent meta-analyses, a sound and reproducible assessment of the current information base was possible. As a result, quantitative and qualitative durable information is now available on contact frequencies, occupational hygiene practice, training efficiency, and consciousness of behaviours relevant to oral exposure. In addition, the currently available measurement data that could be used to evaluate an occupational oral exposure model was identified and systematically summarized.

## Conclusion

To the best of the authors’ knowledge, both systematic reviews and meta-analyses are the first for workplace behaviour and occupational hygiene practices and measurement data on occupational oral exposure. The results indicate that behaviour and hygiene practices need to be taken into account when assessing occupational oral exposure, particularly in terms of contact frequency or the controllability of hand-face contacts. Other parameters influencing behaviour and hygiene practices in the workplace, such as training and its efficiency, require further research in the future. An overarching challenge in this area is the very limited database for adults, especially for non-healthcare workers. However, the meta-analyses of the aggregated data show that some qualitative and quantitative information is available on aspects influencing occupational oral exposure. In addition, there are at least a few studies with measurement data on occupational oral exposure.

## Supplementary material

Supplementary material is available at *Annals of Work Exposures and Health* online.

wxaf015_suppl_Supplementary_Material

## Data Availability

The original contributions presented in the study are included in the article/supplementary material, further inquiries can be directed to the corresponding author.
