# Supplementary material for: Behaviour in the workplace and monitoring data on occupational oral exposure to hazardous substances as prerequisites for the development of a model on occupational oral exposure: a systematic review and meta-analysis
Source: Ann Work Expo Health. 2025 May 2;69(5):461–72. doi: 10.1093/annweh/wxaf015 (PMC12214871; doi:10.1093/annweh/wxaf015)
Supplement: wxaf015_suppl_Supplementary_Material [file wxaf015_suppl_supplementary_material.pdf]

Supplementary Material:

Behaviour in the workplace and monitoring data on occupational oral exposure to hazardous substances as prerequisites for the development of a model on occupational oral exposure: A systematic review and meta-analysis

Marlene Dietz[1,2*], Anke Kahl[2], Urs Schlüter[1]

[1]Unit 4.I.4 Exposure Assessment, Exposure Science, Division 4 Hazardous Substances and Biological Agents, Federal Institute for Occupational Safety and Health (BAuA), D-44149 Dortmund, Germany
[2]Chair of Occupational Safety, School of Mechanical Engineering and Safety Engineering, University of Wuppertal, D-42119 Wuppertal, Germany

*** Correspondence:**
Marlene Dietz
Dietz.Marlene@baua.bund.de

1. Set-up and conduction of the two systematic reviews

Supplementary Table 1: Overview of searched databases and institutional websites.

Information source	Database	Website	URL
bergischbib	x		http://www.bergischbib.de/
COCHRANE	x		https://www.cochranelibrary.com/advanced-search
Deutsche Nationalbibliothek	x		https://katalog.dnb.de
PubMed	x		https://pubmed.ncbi.nlm.nih.gov/advanced/
PubPsych	x		https://pubpsych.de/
Web of Science	x		https://www.webofscience.com
Federal Institute for Occupational Safety and Health (BAuA)		x	https://www.baua.de/DE/Angebote/Publikationen/Publikationen_node.html
United States Environmental Protection Agency (EPA)		x	https://www.epa.gov/nscep (Advanced search)
Health and Safety Executive (HSE)		x	https://www.hse.gov.uk/pubns/
Institute of Occupational Medicine (IOM)		x	https://www.iom-world.org/research/online-library/
National Institute for Occupational Safety and Health (NIOSH)		x	https://www2a.cdc.gov/nioshtic-2/advsearch2.asp
Organisation for Economic Co-operation and Development (OECD)		x	https://www.oecd-ilibrary.org/

National Institute for Public Health and the Environment (RIVM)		x	https://www.rivm.nl/en/recentpublications
Netherlands Organisation for Applied Scientific Research (TNO)		x	https://repository.tno.nl/islandora/search/
World Health Organization (WHO)		x	https://apps.who.int/iris/

Supplementary Table 2: Standard search strategies for each topic.

No	Topic	Standard search strategy
1	Relevant behavior	(profession* OR participant* OR Worker OR office) AND ("contact rate" OR "contact frequenc*" OR Behav* OR self-touch*) AND (peri-oral* OR perioral* OR face) AND (hand OR hands)
2	Hygiene practice	(employee OR Worker OR office) AND ((("hand wash*" OR handwash*) AND (questionnaire OR pract* OR frequenc* OR often OR observ*)) OR ("occupational hygiene" AND (practic* OR observ* OR questionnaire OR implement*))) NOT ("hand wash sampl*")
3	Trainings	(employee OR Worker OR office) AND ("occupational hygiene" OR OSH OR OHS) AND (training) AND (effect* OR improv* OR deteriorat* OR degrad*)
4	Consciousness	touch* AND face AND (reason OR trigger OR awareness)
5	Measurement data	("oral exposure" OR "inadvertent ingestion" OR "inadvertently ingested" OR peri-oral OR perioral OR lips OR saliva OR mouth rinse* OR "ingestion volume" OR "ingestion mass") AND (Occupa* OR Worker OR Workplace OR industr*) AND (measur* OR monitoring OR sampl*) NOT (patient* OR rat OR rats OR mouse OR mice OR pig OR pigs OR SARS-CoV-2 OR COVID-19 OR stress* OR sleep* OR psychologic* OR administration OR drug* OR amalgam* OR cancer OR noise OR "smoking cessation" OR "lip seal*" OR "lip product*")

Supplementary Table 3: Overview of publications used for the evaluation of search strategies.

Search strategy	Evaluation publication	First author	Title	Year	Study
1	1	Gorman Ng	Inadvertent ingestion exposure: hand- and object-to-mouth behavior among workers	2016	**(1)**
1	2	Nicas	A study quantifying the hand to face contact rate and its potential application to predicting respiratory tract infection	2008	**(2)**
1	3	Zhang	Most self touches are with the nondominant hand	2020	**(3)**
1	4	Zivich	Measuring office workplace interactions and hand hygiene behaviors through electronic sensors: A feasibility study	2021	**(4)**

1	5	Mueller	Self-touch: Contact durations and point of touch of spontaneous facial self-touches differ depending on cognitive and emotional load	2019	**(5)**
1	6	Rahman	How frequently do we touch facial TZone A Systematic Review	2020	**(6)**
1	7	Lewis	Hand-to-mouth and other hand-to-face touching behavior in a quasi-naturalistic study under controlled conditions	2021	**(7)**
2	1	Strohbehn	Hand Washing Frequencies and Procedures Used in Retail Food Services	2008	**(8)**
2	2	Vermeulen	Dermal Exposure, Handwashing, and Hand Dermatitis in the Rubber Manufacturing Industry	2001	**(9)**
2	3	Surakka	Workers' Dermal Exposure to UV-curable Acrylates in the Furniture and Parquet Industry	2000	**(10)**
2	4	Albright	Use patterns and frequency of hand hygiene in healthcare facilities Analysis of electronic surveillance data	2018	**(11)**
2	5	Watkins	Exposure to PBDEs in the Office Environment: Evaluating the Relationships between Dust, Handwipes, and Serum	2011	**(12)**
3	1	Robson	A systematic review of the effectiveness of occupational health and safety training	2012	**(13)**
4	1	Spille	Stop touching your face! A systematic review of triggers, characteristics, regulatory functions and neuro-physiology of facial self touch	2021	**(14)**
5	1	Sinclair	Measuring water ingestion from spray exposures	2016	**(17)**
5	2	Connolly	Evaluating Glyphosate Exposure Routes and Their Contribution to Total Body Burden: A Study Among Amenity Horticulturalists	2019	**(18)**
5	3	Hwan	Using structural equation model to explore occupational lead exposure pathways	2002	**(19)**
5	4	Gorman Ng	Field Measurements of Inadvertent Ingestion Exposure to Metals	2017	**(20)**

Supplementary Table 4: Evaluation results for search strategies. Numbers of publications refer to Supplementary Table 3.

**Search strategy**	**Evaluation publication**	**Web of Science**		**PubMed**	
		**Availability**	**Inclusion**	**Availability**	**Inclusion**
1	1	x	x	x	x
1	2	x	x	x	x
1	3	x	x	x	x
1	4	x	x	x	x
1	5	x	x	x	x
1	6	x	x	x	x

1	7	x	x	x	x
2	1	x	x	x	x
2	2	x	x	x	x
2	3	x	x	x	/
2	4	x	x	x	x
2	5	x	x	x	x
3	1	x	x	x	x
4	1	x	x	x	x
5	1	x	x	x	x
5	2	x	x	x	x
5	3	x	x	x	x
5	4	x	x	x	x

Supplementary Table 5: Dates of last search for all search strategies, databases and websites. Simplified search strategies for websites.

Topic	Search strategy	Database / Website	Date of last search
Relevant behavior	1	Web of Science	2023-01-04
Relevant behavior	1	PubMed	2023-01-04
Relevant behavior	behavior; behaviour; contact face; ingestion	IOM	01-04 & 01-05-2023
Relevant behavior	behaviour of worker; hand mouth contact	HSE	2023-01-05
Relevant behavior	Kontakt Gesicht; Verschlucken	BAuA	01-05 & 01-06-2023
Relevant behavior	behavior AND work; behaviour AND work; hand AND mouth AND contact	RIVM	2023-01-06
Relevant behavior	("contact rate" OR "contact frequenc*" OR Behav* OR self-touch*) AND (peri-oral* OR perioral* OR face); contact mouth	TNO	2023-01-06
Relevant behavior	1	EPA	2023-01-06
Relevant behavior	behavior work; behaviour work; hand mouth contact	NIOSH	2023-01-09
Relevant behavior	behavior AND work; behaviour AND work; hand AND face AND contact	WHO	01-09 & 01-11-2023
Relevant behavior	behavior AND work; hand AND face AND contact	OECD	2023-01-11
Relevant behavior	1	COCHRANE	2023-01-13
Relevant behavior	(Kontakt OR Verhalten OR Berührung) AND (Gesicht OR oral) AND (Hand OR Hände)	Deutsche Nationalbibliothek	2023-02-13
Relevant behavior	1	bergischbib	2023-02-13
Relevant behavior	(Kontakt OR Verhalten OR Berührung) AND (Gesicht OR oral) AND (Hand OR Hände)	bergischbib	2023-02-13
Relevant behavior	1	PubPsych	2023-02-13

Hygiene practice	2	Web of Science	2023-01-04
Hygiene practice	2	PubMed	2023-01-04
Hygiene practice	occupational hygiene	IOM	2023-01-05
Hygiene practice	occupational hygiene	HSE	2023-01-05
Hygiene practice	Arbeitshygiene	BAuA	01-05 & 01-06-2023
Hygiene practice	hygiene AND work	RIVM	2023-01-06
Hygiene practice	2; occupational hygiene	TNO	2023-01-06
Hygiene practice	(employee OR Worker OR office) AND ("hand wash*" OR handwash* OR "occupational hygiene")	EPA	2023-01-06
Hygiene practice	occupational-hygiene	NIOSH	2023-01-09
Hygiene practice	occupational hygiene	WHO	2023-01-11
Hygiene practice	"occupational hygiene"	OECD	2023-01-11
Hygiene practice	2	COCHRANE	2023-01-13
Hygiene practice	(Arbeitshygiene) OR (Arbeit* AND (Händewaschen OR "Hände waschen"))	Deutsche Nationalbibliothek	2023-02-13
Hygiene practice	2	bergischbib	2023-02-13
Hygiene practice	(Arbeitshygiene) OR (Arbeit* AND (Händewaschen OR "Hände waschen"))	bergischbib	2023-02-13
Trainings	3	Web of Science	2023-01-04
Trainings	3	PubMed	2023-01-04
Trainings	training effect	IOM	2023-01-05
Trainings	training effect	HSE	2023-01-05
Trainings	Schulung Effekt	BAuA	01-05 & 01-06-2023
Trainings	training AND effect	RIVM	2023-01-06
Trainings	3; training AND effect AND work	TNO	2023-01-06
Trainings	3	EPA	2023-01-06
Trainings	training effect	NIOSH	2023-01-09
Trainings	occupation AND training AND effect	WHO	2023-01-11
Trainings	occupation AND training AND effect	OECD	2023-01-11
Trainings	3	COCHRANE	2023-01-13
Trainings	Arbeitshygiene AND Schulung	Deutsche Nationalbibliothek	2023-02-13
Trainings	3	bergischbib	2023-02-13
Trainings	Arbeitshygiene AND Schulung	bergischbib	2023-02-13
Trainings	3	PubPsych	2023-02-13
Consciousness	4	Web of Science	2023-01-04
Consciousness	4	PubMed	2023-01-04
Consciousness	psychology; touch face; trigger	IOM	2023-01-05
Consciousness	psychology touch	HSE	2023-01-05
Consciousness	Psychologie Berührung; Berührung Gesicht	BAuA	2023-01-06
Consciousness	psychology touch; touch face; trigger	RIVM	2023-01-06
Consciousness	psychology touch; touch face	TNO	2023-01-06
Consciousness	4	EPA	2023-01-06
Consciousness	touch face	NIOSH	2023-01-09
Consciousness	reason AND touch	WHO	2023-01-11
Consciousness	reason AND touch	OECD	2023-01-11
Consciousness	4	COCHRANE	2023-01-13

Consciousness	Berührung AND Gesicht AND Bewusst*	Deutsche Nationalbibliothek	2023-02-13
Consciousness	4	bergischbib	2023-02-13
Consciousness	Berührung AND Gesicht AND Bewusst*	bergischbib	2023-02-13
Consciousness	4	PubPsych	2023-02-13
Measurement data	5	Web of Science	2023-10-02
Measurement data	5	PubMed	2023-10-02
Measurement data	saliva; mouth rinse; perioral; peri-oral; ingestion	IOM	2023-10-02
Measurement data	saliva sample; mouth rinse; perioral; peri-oral; ingestion	HSE	2023-10-02
Measurement data	Speichel; saliva; Mundspülung; mouth rinse; perioral; peri-oral; verschlucken; ingestion	BAuA	2023-10-02
Measurement data	saliva; mouth rinse; perioral; peri-oral; ingestion	RIVM	2023-10-02
Measurement data	("oral exposure" OR "inadvertent ingestion" OR "inadvertently ingested" OR peri-oral OR perioral OR lips OR saliva OR mouth rinse* OR "ingestion volume" OR "ingestion mass") AND (Occupa* OR Worker OR Workplace OR industr*) AND (measur* OR monitoring OR sampl*)	TNO	2023-10-02
Measurement data	("oral exposure" OR "inadvertent ingestion" OR "inadvertently ingested" OR peri-oral OR perioral OR lips OR saliva OR mouth rinse* OR "ingestion volume" OR "ingestion mass") AND (Occupa* OR Worker OR Workplace OR industr*)	EPA	2023-10-02
Measurement data	5	NIOSH	2023-10-02
Measurement data	saliva AND workplace AND measurement NOT SARS-CoV2; mouth rinse AND workplace AND measurement NOT SARS-CoV2; perioral AND workplace AND measurement NOT SARS-CoV2; peri-oral AND workplace AND measurement NOT SARS-CoV2; ingestion AND workplace AND measurement NOT SARS-CoV2	WHO	2023-10-02
Measurement data	5	OECD	2023-10-02
Measurement data	("oral exposure" OR "inadvertent ingestion" OR "inadvertently ingested" OR peri-oral OR perioral OR lips OR saliva OR mouth rinse* OR "ingestion volume" OR "ingestion mass") AND (Occupa* OR Worker OR Workplace OR industr*) AND (measur* OR monitoring OR sampl*) NOT (patient* OR rat OR rats OR mouse OR mice OR pig OR pigs OR SARS-CoV-2 OR COVID-19 OR stress* OR sleep* OR psychologic* OR administration OR drug* OR amalgam* OR cancer OR noise OR	COCHRANE	2023-10-02

	"smoking cessation" OR (lip NEXT seal*) OR (lip NEXT product*))		
Measurement data	("oral exposure" OR "inadvertent ingestion" OR "inadvertently ingested" OR peri-oral OR perioral OR lips OR saliva OR mouth rinse* OR "ingestion volume" OR "ingestion mass") AND (Occupa* OR Worker OR Workplace OR industr*) AND (measur* OR monitoring OR sampl*)	Deutsche Nationalbibliothek	2023-10-02
Measurement data	("orale Exposition" OR peri-oral OR perioral OR Lippen OR Speichel OR Mundspülung) AND (Arbeit* OR Industrie*)	Deutsche Nationalbibliothek	2023-10-02
Measurement data	("oral exposure" OR "inadvertent ingestion" OR "inadvertently ingested" OR peri-oral OR perioral OR lips OR saliva OR mouth rinse* OR "ingestion volume" OR "ingestion mass") AND (Occupa* OR Worker OR Workplace OR industr*) AND (measur* OR monitoring OR sampl*)	bergischbib	2023-10-02

Supplementary Table 6: Population and Outcome criteria for all search strategies for both title and abstract (step 1) and full text (step 2) screening.

Search strategy	Step	Population	Outcome
1. Relevant behavior	1	Adults	Any information on contacts or behaviour which can lead to oral exposure
	2	Adults	Quantitative information on relevant behaviour (frequencies)
2. Occupational hygiene	1	Employees	Any information on occupational hygiene
	2	Employees	Qualitative information on hygiene practices influences by behaviour and not already covered by TRGS 500 (21)<br>Quantitative information: Efficiencies or frequencies of measures
3. Training	1	Employees	Any indication on frequency of trainings or their efficiency regarding change of behaviour or the height of occupational exposure
	2	Employees	Quantitative information on efficiencies except of studies including feedback, audits, modification of work conditions, limitations due to e.g., lack of water, activity specific trainings e.g., welding
4. Consciousness	1	Adults	Hints on avoidability or subconsciousness of behaviour
	2	Adults	Qualitative and quantitative information on controllability of own behaviour
5. Measurement data	1	Employees	Hints on measurement data allowing conclusions on occupational oral exposure
	2	Employees	Concrete measurement data allowing conclusions on occupational oral exposure

Supplementary Table 7: Overview of all 142 included studies from behavioural review with extracted information for the contact frequency, qualitative and quantitative measures, training and consciousness.

			**Coarse categories**					**Groups of workplaces**																	
First author	Year	Study	Contact frequency	Qualitative measures	Quantitative measures	Training	Consciousness	n.a.	Waste	Accumulators	Outdoor	Construction	Energy sector	Indoors	Nursing	Laboratory	Agriculture	Metal working	Food	Polymers	Recycling (e-waste)	Pesticides	Other industries	Weapons	Workshops
Abbas	2020	(22)				x									x										
Akyol	2007	(23)			x												x								
Albright	2018	(11)			x										x										
Aryal	2019	(24)				x																	x		
Bartnicka	2021	(25)				x						x													
Bauer	2018	(26)			x	x																	x		
Beine	2022	(27)			x																				
Berumen-Flucker	2019	(28)				x																			
Blanco	2005	(29)		x							x						x								
Burns	2021	(30)			x							x						x					x		
Cherrie	2006	(31)	x									x													
Christensen	2021	(32)	x				x							x		x						x	x		
Christopher	2007	(33)	x					x																	
Clemmensen	2015	(34)			x	x									x		x	x							
Curwin	2003	(35)			x																		x		

Curwin	2005	(36)			x												x								
das Neves	2006	(37)				x											x								
Deepak	2020	(38)			x										x										
Duran	2013	(39)					x								x										
Durmusoglu	2020	(40)			x			x																	
Elder	2014	(41)	x		x		x																		x
Findik	2011	(42)			x										x										
Fitzpatrick	2011	(43)				x									x										
Gorman Ng	2016	(1)	x				x	x																	
Gould	2017	(44)				x							x			x		x					x		x
Green	2005	(45)			x										x										
Grunwald	2014	(46)					x												x						
Grzywacz	2022	(47)				x								x											
Gu	2015	(48)			x												x								
Hamid	2018	(49)			x																		x		
Hamnerius	2018	(50)			x										x										
Hasylin	2022	(51)		x											x										
Jensen	2015	(52)			x																		x		
Johnston	2014	(53)	x		x														x						
Joshi	2017	(54)				x					x	x													
Karaoglu	2018	(55)				x									x										
Korinth	2007	(56)			x										x										
Kwok	2015	(57)	x								x						x						x		
Lee	2013	(58)			x																				x
Lewis	2021	(7)	x												x										
Liebst	2022	(59)					x																		
Löffler	2006	(60)				x																	x		
Luong Thanh	2016	(61)				x									x										
MacFarlane	2015	(62)			x			x				x			x		x								

Malhotra	2008	(63)				x									x										
Manghisi	2020	(64)					x																x		
Meding	2006	(65)			x			x																	
Mueller	2019	(5)					x																		
Naikoba	2001	(66)				x																	x		
Natnael	2022	(67)			x										x										
Nicas	2008	(2)	x											x											
Nik Husain	2018	(68)				x								x											
Oh	2021	(69)	x											x											
Ozyazicioğlu	2010	(70)			x										x										
Picheansathian	2004	(71)			x										x										
Rahman	2020	(6)	x				x																		
Ralph	2022	(72)	x				x							x											
Roberts	2022	(73)				x													x						
Roman-Muniz	2016	(74)				x									x										
Rosenthal	2003	(75)				x													x						
Sadeghi-Moghaddam	2015	(76)				x									x										
Sharma	2021	(77)			x									x											
Shi	2017	(78)			x									x											
Shiraly	2020	(79)	x				x				x			x											
Soon	2012	(80)				x											x		x						
Spille	2022	(81)	x				x							x											
Spille	2021	(14)					x	x																	
Spille	2022	(82)					x							x											
Steiner	2011	(83)			x														x						
Strohbehn	2008	(8)			x														x						

Surakka	2000	(10)			x																		x		
Szabó	2015	(84)			x										x										
Tang	2022	(85)	x				x							x											
Tao	2020	(86)	x											x											
Teker	2015	(87)				x									x										
Todd	2010	(88)			x														x						
Var	2020	(89)				x									x										
Vermeulen	2001	(9)			x																		x		
Viegas	2022	(90)				x												x							
Visser	2011	(91)			x										x										
Watkins	2011	(12)			x									x											
Wiener	2021	(92)	x				x								x										
Zack	2018	(93)			x									x	x								x		
Zhang	2020	(3)	x											x											
Zivich	2021	(4)			x									x											

Supplementary Table 8: Overview of all nine included studies from measurement data review with extracted information for the perioral wipes, lip wipes, saliva samples / mouth rinses, finger nail scrapings and biomonitoring.

			Sampling					Groups of workplaces				Groups of substances			
**First Author**	Year	Study	Perioral (wipes)	Lips (wipes)	Saliva sample / mouth rinse	Finger nail scraping	Biomonitoring	Agriculture	Metal working	Nursing	Workshop	Metal	Pharmaceuticals	Pesticides	Water
Christopher	2007	(33)	x		x			x	x	x		x	x	x	
Hughson	2004	(94)	x						x			x			
Hughson	2005	(95)	x						x			x			
Hughson	2005	(96)	x			x			x			x			
Connolly	2019	(18)	x					x						x	
Gorman Ng	2017	(20)	x						x			x			
Hwan	2002	(19)		x					x			x			
Hwang	2000	(97)		x					x			x			
Sinclair	2016	(17)					x				x				x

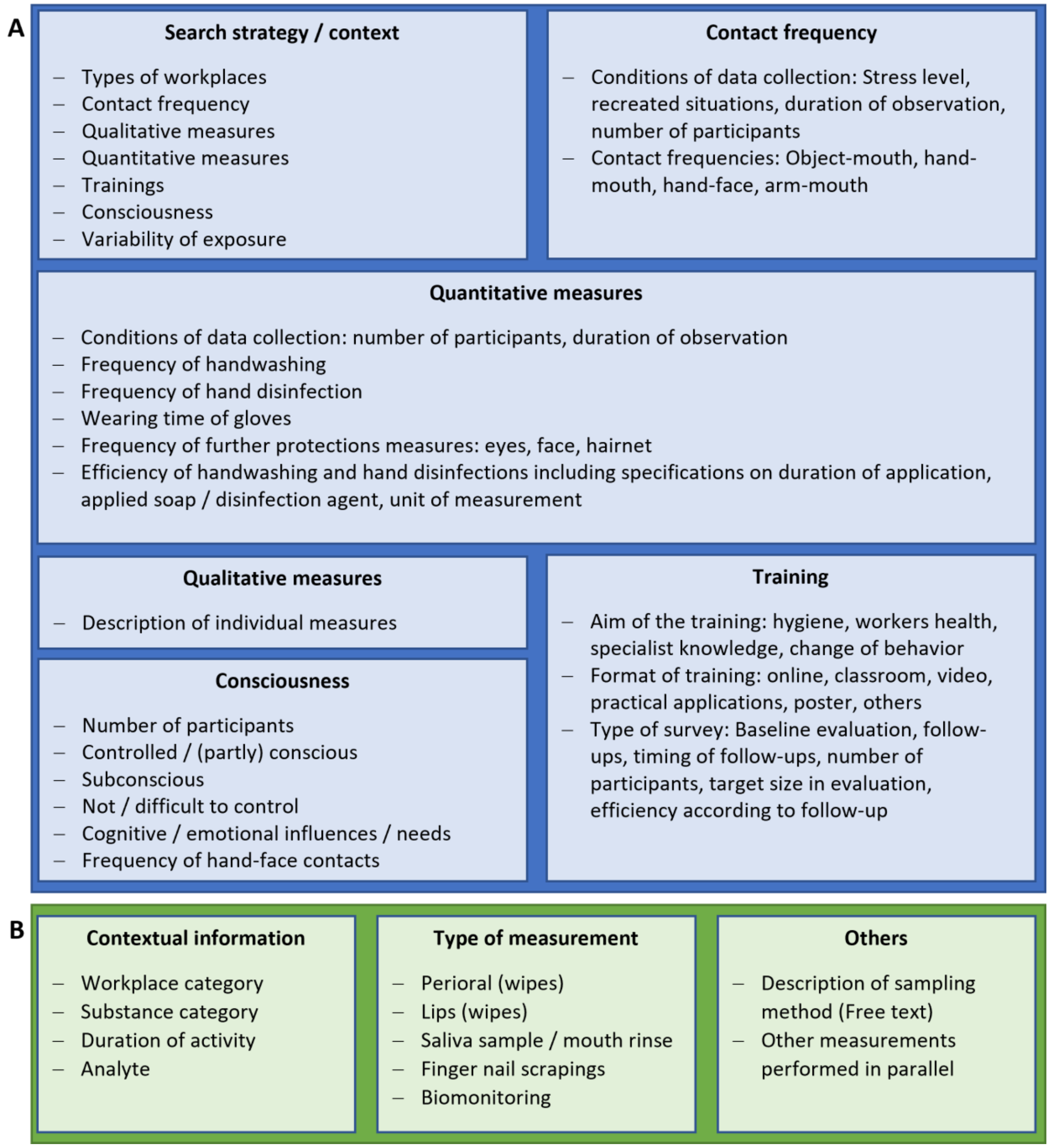

Supplementary Figure 1: Categories grouped by search strategies for review on (A) behaviour and occupational hygiene practice and (B) measurement data.
Alt text: Collection of detailed categories for the data extraction with subfigures labelled from A to B.

2. Calculation of weighted means and Gaussian error propagation for occupational hygiene practices

To summarize these intervals, weighted means $\bar{x}$ were calculated based on the observation duration per interval t_i, the interval center I_i, the corresponding number of workers per interval n_i and the total number of workers per observation $n_{over,obs}$:

$$\bar{x} = \frac{1}{M}\sum_{i=1}^{M}\frac{1}{t_i}\frac{I_i n_i}{n_{over,obs}}$$

Based on the left boundary of the interval $I_{left\ boundary}$ and the interval width $Interval\ width_i$, the interval center I_i is calculated as

$$I_i = I_{left\ boundary} + \frac{Interval\ width_i}{2}$$

The last interval centre (e.g., at least 8 contacts per day) is calculated based on the average of the remaining study interval widths. In our example, with an observation time of one hour, the exemplary calculation is summarized in Table 1.

Table 1: Example calculation of weighted average of handwash frequency. Values in italics are calculated based on other interval values.

Intervals	0-4	5-7	≥ 8
Interval width	4	2	*3*
Interval mean	2	6	*9.5*
Observation duration	1h	1h	1h
Workers	5	3	2
$\bar{x} = \frac{1}{3}\Big[$	$\left(\frac{1}{1h}\frac{2\ cont.\cdot 5\ work.}{10\ work.}\right) +$	$\left(\frac{1}{1h}\frac{6\ cont.\cdot 3\ work.}{10\ work.}\right) +$	$\left(\frac{1}{1h}\frac{9.5\ cont.\cdot 2\ work.}{10\ work.}\right)\Big]$

However, the centre of the interval is not exactly the observed frequency and is therefore error-prone. The maximum deviation between the interval centre and the true frequency $\Delta\overline{x_n}$ is

$$\Delta\bar{x}_i = \pm\frac{1}{2}Interval\ width_i$$

According to a Gaussian error propagation, the total error based on assuming the interval centre as the frequency value is:

$$\Delta\bar{x} = \sqrt{\sum_{i=1}^{M}(\Delta\bar{x}_i)^2\cdot(\frac{\partial\bar{x}}{\partial I_i})^2}$$

with

$$\frac{\partial\bar{x}}{\partial I_i} = \frac{1}{M}\frac{1}{t_i}\frac{1}{n_{over,obs}}n_i$$

the overall frequency calculation results in

$$\bar{x} = \frac{1}{M}\sum_{i=1}^{M}\frac{1}{t_i}\frac{I_i n_i}{n_{over,obs}} \pm \Delta\bar{x}$$

3. Cumulative distribution of hand-face contacts

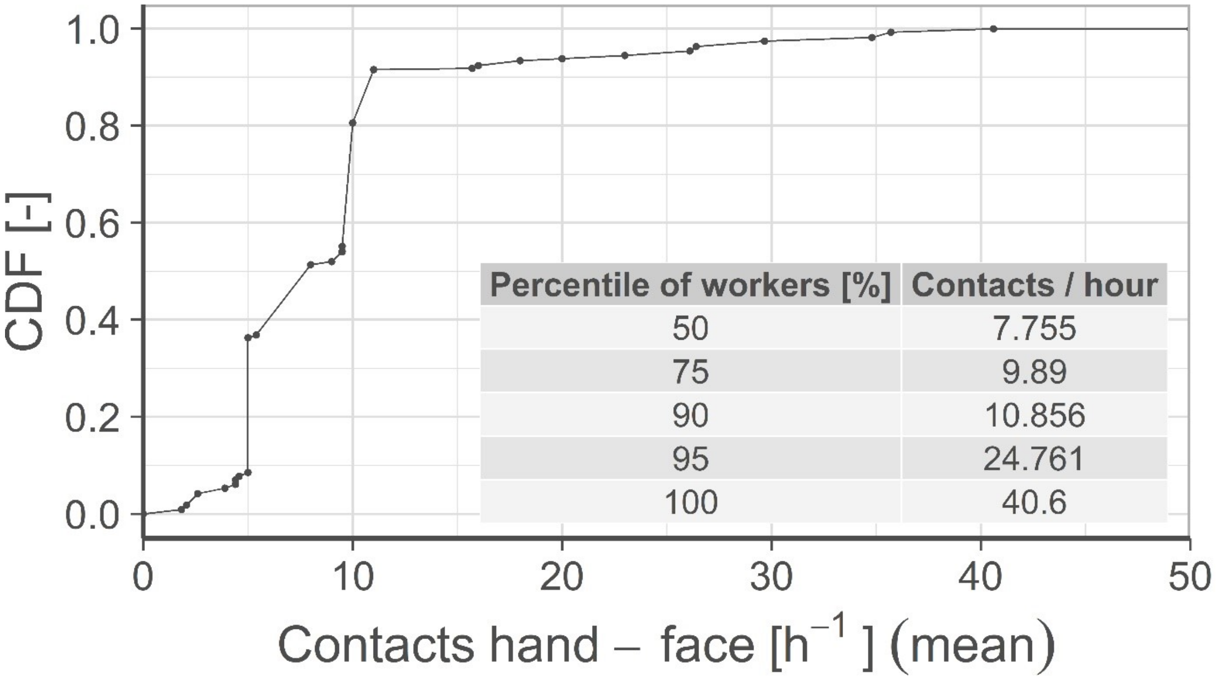

Supplementary Figure 2: Cumulative distribution of hand-face contact based on published mean values and corresponding percentile values based on number of workers. The Poisson distribution for hand-face contacts per hour follows λ = E(X)=Var(X) = 9.279 h^{-1}.
Alt text: Chart depicting the cumulative distribution of hand-face contacts per hour with exemplary percentiles of workers and corresponding contact frequencies.

4. Identified data from measurement methods

Supplementary Table 9: Number of studies and measurement scenarios (sce.) on specific substances and corresponding sampling approaches which include measurement data of occupational oral exposure.

	**Metals**		**Pesticides**		**Pharmaceuticals**		**Water**		**Overall**	
	Studies	Sce.	Studies	Sce.	Studies	Sce.	Studies	Sce.	Studies	Sce.
**Salvia sample / mouth rinse**	**1**	**5**	**1**	**8**	**1**	**6**	**0**	**0**	**1**	**19**
**Perioral (wipes)**	**4**	**304**	**1**	**16**	**1**	**6**	**0**	**0**	**6**	**326**
**Lips (wipes)**	**2**	**8**	**0**	**0**	**0**	**0**	**0**	**0**	**2**	**8**
**Finger nail scraping**	**1**	**64**	**0**	**0**	**0**	**0**	**0**	**0**	**1**	**64**
**Biomonitoring**	**0**	**0**	**0**	**0**	**0**	**0**	**1**	**2**	**1**	**2**

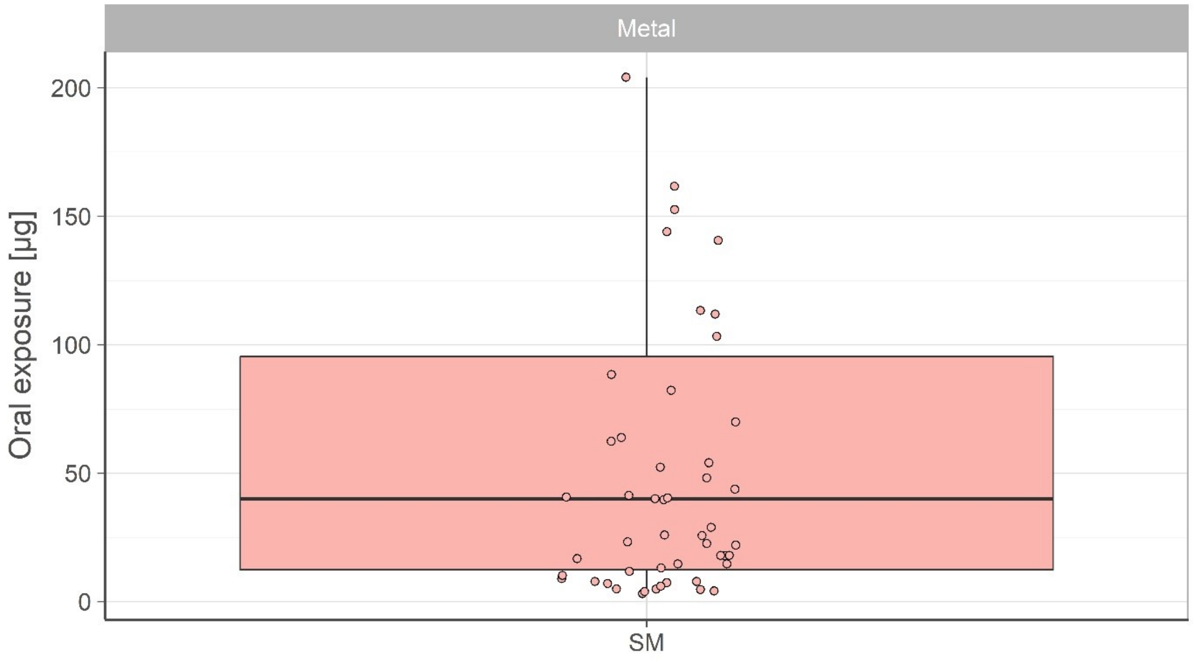

Supplementary Figure 3: Boxplot of the available measurement data on oral exposure based on finger nail scrapings. Outliers are not plotted for better readability. SM: single measurements.
Alt text: Boxplot representing measurement data on occupational oral exposure for finger nail scrapings.

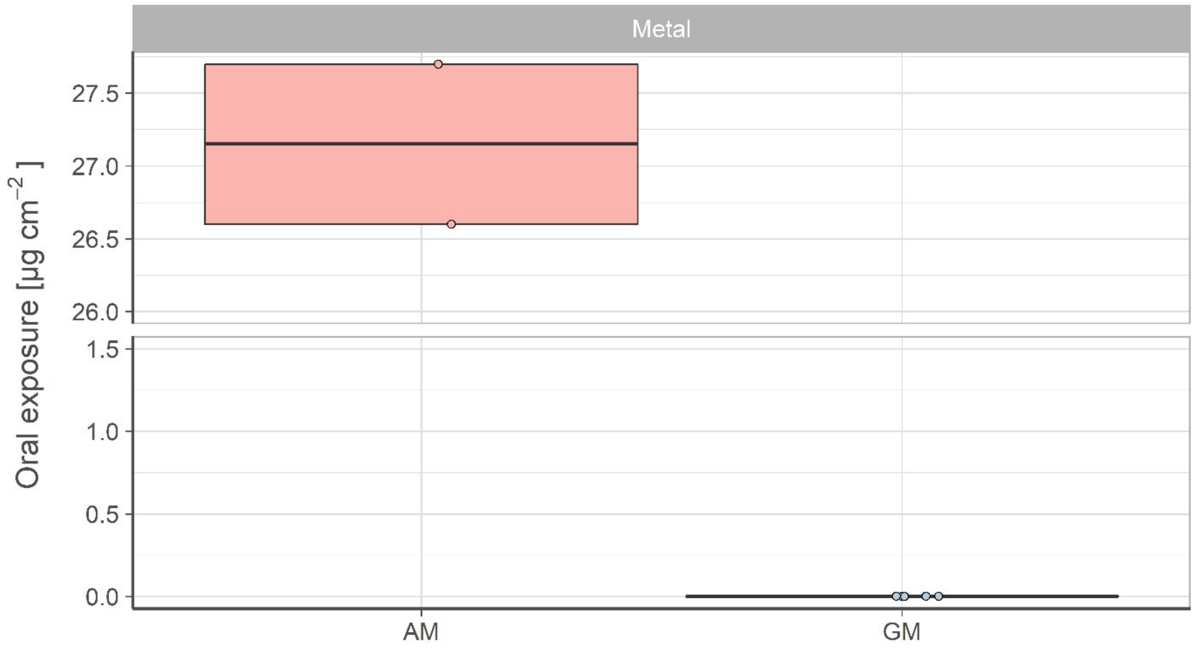

Supplementary Figure 4: Boxplot of the available measurement data on oral exposure based on lip wipes. Outliers are not plotted for better readability.
Alt text: Boxplot representing measurement data on occupational oral exposure for lip wipes.

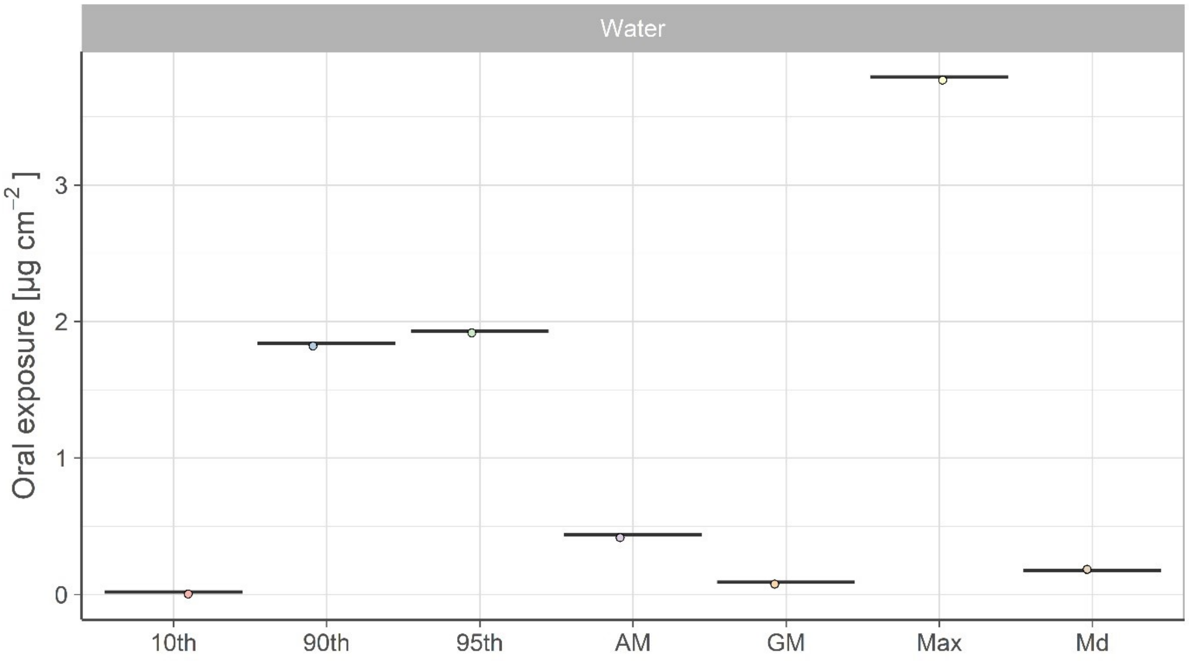

Supplementary Figure 5: Boxplot of the available measurement data on oral exposure based on biomonitoring. Outliers are not plotted for better readability.
Alt text: Boxplot representing measurement data on occupational oral exposure for biomonitoring.

References of included and evaluated studies of both systematic reviews

1. Gorman Ng M, Davis A, van Tongeren M, Cowie H, Semple S. Inadvertent ingestion exposure: hand- and object-to-mouth behavior among workers. J Expo Sci Environ Epidemiol. 2016;26(1):9-16.
2. Nicas M, Best D. A study quantifying the hand-to-face contact rate and its potential application to predicting respiratory tract infection. J Occup Environ Hyg. 2008;5(6):347-52.
3. Zhang N, Jia W, Wang P, King MF, Chan PT, Li Y. Most self-touches are with the nondominant hand. Sci Rep. 2020;10(1):10457.
4. Zivich PN, Huang W, Walsh A, Dutta P, Eisenberg M, Aiello AE. Measuring office workplace interactions and hand hygiene behaviors through electronic sensors: A feasibility study. PLOS ONE. 2021;16(1):e0243358.
5. Mueller SM, Martin S, Grunwald M. Self-touch: Contact durations and point of touch of spontaneous facial self-touches differ depending on cognitive and emotional load. PLOS ONE. 2019;14(3):e0213677.
6. Rahman J, Mumin J, Fakhruddin B. How Frequently Do We Touch Facial T-Zone: A Systematic Review. Annals of Global Health. 2020;86.
7. Lewis RC, Rauschenberger R, Kalmes R. Hand-to-mouth and other hand-to-face touching behavior in a quasi-naturalistic study under controlled conditions. J Toxicol Environ Health A. 2021;84(2):49-55.
8. Strohbehn C, Sneed J, Paez P, Meyer J. Hand washing frequencies and procedures used in retail food services. J Food Prot. 2008;71(8):1641-50.
9. Vermeulen R, Kromhout H, Bruynzeel DP, de Boer EM, Brunekreef B. Dermal exposure, handwashing, and hand dermatitis in the rubber manufacturing industry. Epidemiology. 2001;12(3):350-4.
10. Surakka J, Lindh T, Rosén G, Fischer T. Workers' dermal exposure to UV-curable acrylates in the furniture and parquet industry. The Annals of Occupational Hygiene. 2000;44(8):635-44.
11. Albright J, White B, Pedersen D, Carlson P, Yost L, Littau C. Use patterns and frequency of hand hygiene in healthcare facilities: Analysis of electronic surveillance data. Am J Infect Control. 2018;46(10):1104-9.
12. Watkins DJ, McClean MD, Fraser AJ, Weinberg J, Stapleton HM, Sjödin A, et al. Exposure to PBDEs in the office environment: evaluating the relationships between dust, handwipes, and serum. Environ Health Perspect. 2011;119(9):1247-52.
13. Robson LS, Stephenson CM, Schulte PA, Amick BC, 3rd, Irvin EL, Eggerth DE, et al. A systematic review of the effectiveness of occupational health and safety training. Scand J Work Environ Health. 2012;38(3):193-208.
14. Spille JL, Grunwald M, Martin S, Mueller SM. Stop touching your face! A systematic review of triggers, characteristics, regulatory functions and neuro-physiology of facial self touch. Neuroscience & Biobehavioral Reviews. 2021;128:102-16.
15. Symanski E, Maberti S, Chan W. A meta-analytic approach for characterizing the within-worker and between-worker sources of variation in occupational exposure. Ann Occup Hyg. 2006;50(4):343-57.
16. Kromhout H, Vermeulen R. Temporal, personal and spatial variability in dermal exposure. Ann Occup Hyg. 2001;45(4):257-73.
17. Sinclair M, Roddick F, Nguyen T, O'Toole J, Leder K. Measuring water ingestion from spray exposures. Water Res. 2016;99:1-6.
18. Connolly A, Coggins MA, Galea KS, Jones K, Kenny L, McGowan P, et al. Evaluating Glyphosate Exposure Routes and Their Contribution to Total Body Burden: A Study Among Amenity Horticulturalists. Ann Work Expo Health. 2019;63(2):133-47.
19. Hwan YH, Chang CW, Chao KY, Hsiao FT, Chang HL, Han HZ. Using structural equation model to explore occupational lead exposure pathways. Sci Total Environ. 2002;284(1-3):95-108.
20. Gorman Ng M, MacCalman L, Semple S, van Tongeren M. Field Measurements of Inadvertent Ingestion Exposure to Metals. Ann Work Expo Health. 2017;61(9):1097-107.

21. Committee on Hazardous Substances (AGS). Technische Regeln für Gefahrstoffe. Schutzmaßnahmen. TRGS 500. https://www.baua.de/DE/Angebote/Regelwerk/TRGS/TRGS.html2019.
22. Abbas K, Nawaz SMA, Amin N, Soomro FM, Abid K, Ahmed M, et al. A web-based health education module and its impact on the preventive practices of health-care workers during the COVID-19 pandemic. Health Educ Res. 2020;35(5):353-61.
23. Akyol AD. Hand hygiene among nurses in Turkey: opinions and practices. J Clin Nurs. 2007;16(3):431-7.
24. Aryal A, Parish M, Rohlman DS. Generalizability of Total Worker Health® Online Training for Young Workers. International Journal of Environmental Research and Public Health. 2019;16(4):577.
25. Bartnicka J, Kabiesz P, Palka D, Gajewska P, Islam EU, Szymanek D. Evaluation of the Effectiveness of Employers and H&S Services in Relation to the COVID-19 System in Polish Manufacturing Companies. Int J Environ Res Public Health. 2021;18(17).
26. Bauer A, Rönsch H, Elsner P, Dittmar D, Bennett C, Schuttelaar MLA, et al. Interventions for preventing occupational irritant hand dermatitis. Cochrane Database Syst Rev. 2018;4(4):Cd004414.
27. Beine A, Gina M, Hoffmeyer F, Lotz A, Nöllenheidt C, Zahradnik E, et al. Skin symptoms in veterinary assistant staff and veterinarians: A cross-sectional study. Contact Dermatitis. 2022;87(3):247-57.
28. Berumen-Flucker B, Rodriguez A, Cienega L, Casanova V, Pompeii L, Gimeno Ruiz de Porras D, et al. Evaluation of Safety Management and Leadership Training Using Mobile Technologies among Logging Supervisors. J Agromedicine. 2019;24(2):197-204.
29. Blanco LE, Aragón A, Lundberg I, Lidén C, Wesseling C, Nise G. Determinants of Dermal Exposure among Nicaraguan Subsistence Farmers during Pesticide Applications with Backpack Sprayers. The Annals of Occupational Hygiene. 2005;49(1):17-24.
30. Burns ES, Pathmarajah P, Muralidharan V. Physical and psychological impacts of handwashing and personal protective equipment usage in the COVID-19 pandemic: A UK based cross-sectional analysis of healthcare workers. Dermatol Ther. 2021;34(3):e14885.
31. Cherrie JW, Semple S, Christopher Y, Saleem A, Hughson GW, Philips A. How Important is Inadvertent Ingestion of Hazardous Substances at Work? The Annals of Occupational Hygiene. 2006;50(7):693-704.
32. Christensen PA, Anton JR, Anton CR, Schwartz MR, Anton RC. The role of facial contact in infection control: Renewed import in the age of coronavirus. Am J Infect Control. 2021;49(6):663-73.
33. Christopher Y. Inadvertent ingestion exposure to hazardous substances in the workplace: University of Aberdeen; 2008.
34. Clemmensen KK, Randbøll I, Ryborg MF, Ebbehøj NE, Agner T. Evidence-based training as primary prevention of hand eczema in a population of hospital cleaning workers. Contact Dermatitis. 2015;72(1):47-54.
35. Curwin BD, Hein MJ, Sanderson WT, Nishioka M, Buhler W. Acephate exposure and decontamination on tobacco harvesters' hands. Journal of Exposure Science & Environmental Epidemiology. 2003;13(3):203-10.
36. Curwin BD, Hein MJ, Sanderson WT, Nishioka MG, Buhler W. Nicotine exposure and decontamination on tobacco harvesters' hands. Ann Occup Hyg. 2005;49(5):407-13.
37. das Neves ZC, Tipple AF, Silva e Souza AC, Pereira MS, Melo Dde S, Ferreira LR. Hand hygiene: The impact of incentive strategies on adherence among healthcare workers from a newborn intensive care unit. Rev Lat Am Enfermagem. 2006;14(4):546-52.
38. Deepak, Faujdar SS, Kumar S, Mehrishi P, Solanki S, Sharma A, et al. Hand hygiene knowledge, attitude, practice and hand microflora analysis of staff nurses in a rural tertiary care hospital. J Family Med Prim Care. 2020;9(9):4969-73.
39. Duran ND, Dale R, Kello CT, Street CN, Richardson DC. Exploring the movement dynamics of deception. Front Psychol. 2013;4:140.
40. Durmuşoğlu H, Incili G, Demir P, Ilhak O. Effects of workers' hand washing and knife disinfection. Journal of Food Process Engineering. 2020;2020;00:e14918.

41. Elder NC, Sawyer W, Pallerla H, Khaja S, Blacker M. Hand hygiene and face touching in family medicine offices: a Cincinnati Area Research and Improvement Group (CARInG) network study. J Am Board Fam Med. 2014;27(3):339-46.
42. Findik UY, Otkun MT, Erkan T, Sut N. Evaluation of handwashing behaviors and analysis of hand flora of intensive care unit nurses. Asian Nurs Res (Korean Soc Nurs Sci). 2011;5(2):99-107.
43. Fitzpatrick M, Everett-Thomas R, Nevo I, Shekhter I, Rosen LF, Scheinman SR, et al. A novel educational programme to improve knowledge regarding health care-associated infection and hand hygiene. Int J Nurs Pract. 2011;17(3):269-74.
44. Gould DJ, Moralejo D, Drey N, Chudleigh JH, Taljaard M. Interventions to improve hand hygiene compliance in patient care. Cochrane Database Syst Rev. 2017;9(9):Cd005186.
45. Green L, Selman C, Banerjee A, Marcus R, Medus C, Angulo FJ, et al. Food service workers' self-reported food preparation practices: an EHS-Net study. Int J Hyg Environ Health. 2005;208(1-2):27-35.
46. Grunwald M, Weiss T, Mueller S, Rall L. EEG changes caused by spontaneous facial self-touch may represent emotion regulating processes and working memory maintenance. Brain Res. 2014;1557:111-26.
47. Grzywacz JG, Gonzales-Backen M, Liebman A, Trejo M, Ordaz Gudino C, Trejo M, et al. Comparative Effectiveness of Training Alternatives for the EPA's Worker Protection Standard Regulation Among Immigrant Latino Farmworkers. J Occup Environ Med. 2022;64(2):140-5.
48. Gu J, Zhong Y, Hao Y, Zhou D, Tsui H, Hao C, et al. Preventive Behaviors and Mental Distress in Response to H1N1 Among University Students in Guangzhou, China. Asia Pacific Journal of Public Health. 2015;27(2):NP1867-NP79.
49. Hamid KM, Yusuf I, Onoja BA, Koki AY. Knowledge, attitude and practice of medical laboratory practitioners in the fight against Ebola virus disease. J Virus Erad. 2018;4(1):1-5.
50. Hamnerius N, Svedman C, Bergendorff O, Björk J, Bruze M, Pontén A. Wet work exposure and hand eczema among healthcare workers: a cross-sectional study. Br J Dermatol. 2018;178(2):452-61.
51. Hasylin H, Abdul-Mumin KH, Pg-Hj-Ismail P-K, Trivedi A, Win KN. A Preliminary Assessment of Health and Safety in the Automobile Industry in Brunei Darussalam: Workers’ Knowledge and Practice of Organic Solvents. International Journal of Environmental Research and Public Health. 2022;19(23):15469.
52. Jensen DA, Danyluk MD, Harris LJ, Schaffner DW. Quantifying the effect of hand wash duration, soap use, ground beef debris, and drying methods on the removal of Enterobacter aerogenes on hands. J Food Prot. 2015;78(4):685-90.
53. Johnston JD, Eggett D, Johnson MJ, Reading JC. The Influence of Risk Perception on Biosafety Level-2 Laboratory Workers' Hand-To-Face Contact Behaviors. Journal of Occupational and Environmental Hygiene. 2014;11(9):625-32.
54. Joshi S, Amatya P, Poudel B, Yadav SA. Handwashing Practices in Neonatal Intensive Care Unit, Paediatric Intensive Care Unit and Neonatal Nurseries in Patan Hospital. J Nepal Health Res Counc. 2017;15(1):56-60.
55. Karaoglu MK, Akin S. Effectiveness of Hygienic Hand Washing Training on Hand Washing Practices and Knowledge: A Nonrandomized Quasi-Experimental Design. J Contin Educ Nurs. 2018;49(8):360-71.
56. Korinth G, Weiss T, Penkert S, Schaller KH, Angerer J, Drexler H. Percutaneous absorption of aromatic amines in rubber industry workers: impact of impaired skin and skin barrier creams. Occup Environ Med. 2007;64(6):366-72.
57. Kwok YL, Gralton J, McLaws ML. Face touching: a frequent habit that has implications for hand hygiene. Am J Infect Control. 2015;43(2):112-4.
58. Lee SW, Cheong SH, Byun JY, Choi YW, Choi HY. Occupational hand eczema among nursing staffs in Korea: Self-reported hand eczema and contact sensitization of hospital nursing staffs. J Dermatol. 2013;40(3):182-7.
59. Liebst LS, Ejbye-Ernst P, de Bruin M, Thomas J, Lindegaard MR. Face-touching behaviour as a possible correlate of mask-wearing: A video observational study of public place incidents during the COVID-19 pandemic. Transbound Emerg Dis. 2022;69(3):1319-25.

60. Löffler H, Bruckner T, Diepgen T, Effendy I. Primary prevention in health care employees: a prospective intervention study with a 3-year training period. Contact Dermatitis. 2006;54(4):202-9.
61. Luong Thanh BY, Laopaiboon M, Koh D, Sakunkoo P, Moe H. Behavioural interventions to promote workers' use of respiratory protective equipment. Cochrane Database of Systematic Reviews. 2016(12).
62. MacFarlane E, LaMontagne AD, Driscoll T, Nixon RL, Keegel T. Use of antiseptic hand rubs in the health and community services industry: an Australian population-based survey. Contact Dermatitis. 2015;73(3):157-62.
63. Malhotra R, Lal P, Krishna Prakash S, Daga MK, Kishore J. Evaluation of a Health Education Intervention on Knowledge and Attitudes of Food Handlers Working in a Medical College in Delhi, India. Asia Pacific Journal of Public Health. 2008;20(4):277-86.
64. Manghisi VM, Fiorentino M, Boccaccio A, Gattullo M, Cascella GL, Toschi N, et al. A Body Tracking-Based Low-Cost Solution for Monitoring Workers' Hygiene Best Practices during Pandemics. Sensors (Basel). 2020;20(21).
65. Meding B, Wrangsjö K, Hosseiny S, Andersson E, Hagberg S, Torén K, et al. Occupational skin exposure and hand eczema among dental technicians-need for improved prevention. Scand J Work Environ Health. 2006;32(3):219-24.
66. Naikoba S, Hayward A. The effectiveness of interventions aimed at increasing handwashing in healthcare workers - A systematic review. The Journal of hospital infection. 2001;47:173-80.
67. Natnael T, Adane M, Goraw S. Hand hygiene practices during the COVID-19 pandemic and associated factors among barbers and beauty salon workers in Ethiopia. PLoS One. 2022;17(7):e0269225.
68. Nik Husain N, Manan W, Nj N, Nh N, Abdul Rahman R. The Effect of Food Safety Education on Handwashing Practices in School Canteens' Food Handlers. Sains Malaysiana. 2018;47:2119-28.
69. Oh HS, Ryu M, Yang Y. Hand-to-face contact behaviors during indoor activities in daily life among Korean adults: an observational pilot study using videotaping. Epidemiol Health. 2021;43:e2021030.
70. Ozyazicioğlu N, Sürenler S, Tanriverdi G. Hand dermatitis among paediatric nurses. J Clin Nurs. 2010;19(11-12):1597-603.
71. Picheansathian W. A systematic review on the effectiveness of alcohol-based solutions for hand hygiene. Int J Nurs Pract. 2004;10(1):3-9.
72. Ralph F, Large DR, Burnett G, Lang A, Morris A. U can't touch this! Face touching behaviour whilst driving: implications for health, hygiene and human factors. Ergonomics. 2022;65(7):943-59.
73. Roberts KR, Paez P, Sauer K, Alcorn M, Johnson DE. Impact of Training on Employees' Handwashing Behaviors in School Nutrition Programs. J Acad Nutr Diet. 2022;123(5):770-82.e4.
74. Roman-Muniz I, Ahola J, Chahine M, Adams A. Effect of Dairy Beef Quality Assurance Training on Dairy Worker Knowledge and Welfare-Related Practices. Journal of Extension. 2016;54.
75. Rosenthal VD, McCormick RD, Guzman S, Villamayor C, Orellano PW. Effect of education and performance feedback on handwashing: the benefit of administrative support in Argentinean hospitals. Am J Infect Control. 2003;31(2):85-92.
76. Sadeghi-Moghaddam P, Arjmandnia M, Shokrollahi M, Aghaali M. Does training improve compliance with hand hygiene and decrease infections in the neonatal intensive care unit? A prospective study. J Neonatal Perinatal Med. 2015;8(3):221-5.
77. Sharma M, Batra K, Davis RE, Wilkerson AH. Explaining Handwashing Behavior in a Sample of College Students during COVID-19 Pandemic Using the Multi-Theory Model (MTM) of Health Behavior Change: A Single Institutional Cross-Sectional Survey. Healthcare (Basel). 2021;9(1).
78. Shi W, Guo J, Zhou Y, Deng D, Han Z, Zhang X, et al. Phthalate Esters on Hands of Office Workers: Estimating the Influence of Touching Surfaces. Environmental Science & Technology Letters. 2017;4(1):1-5.
79. Shiraly R, Shayan Z, McLaws ML. Face touching in the time of COVID-19 in Shiraz, Iran. Am J Infect Control. 2020;48(12):1559-61.
80. Soon JM, Baines RN. Food safety training and evaluation of handwashing intention among fresh produce farm workers. Food Control. 2012;23(2):437-48.

81. Spille JL, Grunwald M, Martin S, Mueller SM. The suppression of spontaneous face touch and resulting consequences on memory performance of high and low self-touching individuals. Scientific Reports. 2022;12(1):8637.
82. Spille JL, Mueller SM, Martin S, Grunwald M. Cognitive and emotional regulation processes of spontaneous facial self-touch are activated in the first milliseconds of touch: Replication of previous EEG findings and further insights. Cogn Affect Behav Neurosci. 2022;22(5):984-1000.
83. Steiner MF, Dick FD, Scaife AR, Semple S, Paudyal P, Ayres JG. High prevalence of skin symptoms among bakery workers. Occup Med (Lond). 2011;61(4):280-2.
84. Szabó R, Morvai J, Bellissimo-Rodrigues F, Pittet D. Use of hand hygiene agents as a surrogate marker of compliance in Hungarian long-term care facilities: first nationwide survey. Antimicrob Resist Infect Control. 2015;4:32.
85. Tang J, Wang L, Luo T, Wu S, Wu Z, Chen J, et al. Effectiveness of a Brief Mindfulness-Based Intervention of "STOP touching your face" During the COVID-19 Pandemic: a Randomized Controlled Trial. Mindfulness (N Y). 2022;13(12):3123-33.
86. Tao ZY, Dong J, Culleton R. The use of facemasks may not lead to an increase in hand-face contact. Transbound Emerg Dis. 2020;67(6):3038-40.
87. Teker B, Ogutlu A, Gozdas HT, Ruayercan S, Hacialioglu G, Karabay O. Factors Affecting Hand Hygiene Adherence at a Private Hospital in Turkey. Eurasian J Med. 2015;47(3):208-12.
88. Todd EC, Michaels BS, Holah J, Smith D, Greig JD, Bartleson CA. Outbreaks where food workers have been implicated in the spread of foodborne disease. Part 10. Alcohol-based antiseptics for hand disinfection and a comparison of their effectiveness with soaps. J Food Prot. 2010;73(11):2128-40.
89. Var C, Oberhelman RA, Shu T, Leang S, Duggal R, Le J, et al. A Linked Community and Health Facility Intervention to Improve Newborn Health in Cambodia: the NICCI Stepped-Wedge Cluster-Randomized Controlled Trial. Int J Environ Res Public Health. 2020;17(5).
90. Viegas S, Martins C, Bocca B, Bousoumah R, Duca RC, Galea KS, et al. HBM4EU Chromates Study: Determinants of Exposure to Hexavalent Chromium in Plating, Welding and Other Occupational Settings. International Journal of Environmental Research and Public Health. 2022;19(6):3683.
91. Visser MJ, Behroozy A, Verberk MM, Semple S, Kezic S. Quantification of wet-work exposure in nurses using a newly developed wet-work exposure monitor. Ann Occup Hyg. 2011;55(7):810-6.
92. Wiener R, Trickett-Shockey A, Waters C, Bhandari R. Face-touching Behavior during the COVID-19 Pandemic: Self-inoculation and transmission potentials. Journal of dental hygiene: JDH / American Dental Hygienists' Association. 2021;95:41-6.
93. Zack B, Arrandale V, Holness DL. Skin-specific training experience of workers assessed for contact dermatitis. Occup Med (Lond). 2018;68(3):203-6.
94. Hughson GW. An occupational hygiene assessment of dermal nickel exposures in primary production industries. Edinburgh: Institute of Occupational Medicine; 2004.
95. Hughson GW. An occupational hygiene assessment of dermal nickel exposures in primary production and primary user industries. Phase 2 Report. Edinburgh: Institute of Occupational Medicine; 2005.
96. Hughson GW. An occupational hygiene assessment of dermal inorganic lead exposures in primary and intermediate user industries. Edinburgh: Institute of Occupational Medicine; 2005.
97. Hwang YH, Chao KY, Chang CW, Hsiao FT, Chang HL, Han HZ. Lip lead as an alternative measure for lead exposure assessment of lead battery assembly workers. Aihaj. 2000;61(6):825-31.
